# In Vitro and In Silico Evaluations of the Antileishmanial Activities of New Benzimidazole-Triazole Derivatives

**DOI:** 10.3390/vetsci10110648

**Published:** 2023-11-09

**Authors:** Mustafa Eser, İbrahim Çavuş

**Affiliations:** 1 Health Programs, Faculty of Open Education, Anadolu University, Eskisehir 26470, Turkey; 2 Department of Parasitology, Faculty of Medicine, Manisa Celal Bayar University, Manisa 45030, Turkey; ibrahim.cavus@cbu.edu.tr

**Keywords:** antileishmanial activity, leishmaniasis, benzimidazole, triazole, XTT

## Abstract

**Simple Summary:**

Leishmaniasis is a protozoan disease seen in many vertebrates, caused by 20 different Leishmania species, and transmitted by the bite of flies, also known as vector sandflies. Leishmaniasis shows clinical changes such as subclinical, localized (cutaneous leishmaniasis), or widespread infection (cutaneous, mucosal, or visceral), depending on the parasite and host factors. Poor vector (sandfly) control, a lack of immunizations, restricted access to new and affordable medications, and ineffective drug research efforts are the current challenges to effective prevention and management. Drugs used in leishmaniasis chemotherapy may have nephrotoxic, hepatotoxic, and teratogenic side effects. Although many drug combinations have been developed for treatment, new drug formulations are needed due to the emergence of resistance. Some procedures need to be carried out to find new formulations. In this study, the synthesis of the new benzimidazole-triazole therapy, which is frequently used in the structure of drug molecules, is examined, and its antileishmanial activities are evaluated.

**Abstract:**

Benzimidazole and triazole rings are important pharmacophores, known to exhibit various pharmacological activities in drug discovery. In this study, it was purposed to synthesize new benzimidazole-triazole derivatives and evaluate their antileishmanial activities. The targeted compounds (**5a**–**5h**) were obtained after five chemical reaction steps. The structures of the compounds were confirmed by spectral data. The possible in vitro antileishmanial activities of the synthesized compounds were evaluated against the *Leishmania tropica* strain. Further, molecular docking and dynamics were performed to identify the probable mechanism of activity of the test compounds. The findings revealed that compounds **5a**, **5d**, **5e**, **5f**, and **5h** inhibited the growth of *Leishmania tropica* to various extents and had significant anti-leishmanial activities, even if some orders were higher than the reference drug Amphotericin B. On the other hand, compounds **5b**, **5c**, and **5g** were found to be ineffective. Additionally, the results of in silico studies have presented the existence of some interactions between the compounds and the active site of sterol 14-alpha-demethylase, a biosynthetic enzyme that plays a critical role in the growth of the parasite. Therefore, it can be suggested that if the results obtained from this study are confirmed with in vivo findings, it may be possible to obtain some new anti-leishmanial drug candidates.

## 1. Introduction

Leishmaniasis is a parasitic disease sourced in humans by more than 20 types of protozoon parasites [1]. The types of parasites causing the disease are found in the Protista kingdom, the *Trypanosomatidae* family, and the Leishmania genus. Many of them are zoonotic [2]. Parasites of the Leishmania type are spread through the bite of Phlebotomus and Lutzomyia flies, also known as sand flies [3]. More than 90 sandfly species are recognized as leading to infection in living creatures, which can occur in three different forms. The highly fatal form of Visceral Leishmaniasis is also defined as Kala-azar, with an estimated 50,000–90,000 cases of VL reported worldwide each year. Most events arise in India, Brazil, and East Africa. Cases are fatal if left untreated in more than 95% of cases. CL is the other form of the infection, which causes lifelong ulcerative skin lesions of the body. This form is the most common. It is estimated that 600,000–1 million new cases happen annually in the world. Approximately 95% of cases appear in the American Continent, the Mediterranean District, Central Asia, and Middle Eastern countries. ML, which destroys the mucosa in the oral cavity, nose, pharynx, and larynx, is found in Bolivia, Brazil, Ethiopia, and Peru. Increases in disease cases are brought on by numerous risk factors. Poor socioeconomic situations, starvation, inconsistent population movement, and environmental and climate change are some of these threats [1].

Sterol biosynthesis holds a key role for eukaryotic organisms [4,5]. Sterols, which define the membrane liquidity and permeability and regulate the activity of ion channels, are membrane-bound proteins that are necessary in order to form a living eukaryotic layer [6]. Furthermore, they are administered as precursors for biologically dynamic molecules that regulate growth and evolvement durations at a nanomolar concentration [7,8]. Several reports show that functional sterols are important in *Trypanosomatidae* [9,10,11]. Sterol 14-alpha-demethylase, a member of the cytochrome P450 enzyme family, has an essential role in the sterol biosynthetic pathway. The demethylation of the ring system is its main purpose in the lanosterol pathway [12]. Because of the buildup of 14-methylated sterols, the suppression of sterol 14-alpha-demethylase disrupts the stability of the parasites’ membranes and prevents parasite proliferation. [13]. The main benefit of using sterol 14-alpha-demethylase as a therapeutic target against protozoa is that its inhibitors, imidazole and triazole derivatives, are already effective antifungal medications in both clinical and agricultural settings [14,15]. The action of azoles is increased by the accumulation of toxic methylated sterols, which stop fungal growth and cause cell death, in addition to inhibiting sterol production. Numerous investigations have demonstrated that antifungal azoles have an antiparasitic effect on *Trypanosomatidae* cells [16,17,18,19,20,21,22]. Azoles damage the parasite membranes, disturb division and multiplication, and intensely change sterol composition [23,24,25,26].

The lack of an adequate number of effective agents against leishmaniasis demands research on new vaccines or drugs. In addition, the drugs used in the chemotherapy of leishmaniasis can include nephrotoxic, hepatotoxic, and teratogenic side effects. Additionally, cases that develop resistance to antileishmanial drugs due to long-term use lead researchers to work towards producing new drug candidates. For this reason, within the scope of the present study, eight new compounds, containing benzimidazole (BZI) and triazole structures that commonly exist in antiparasitic drugs, were synthesized, and their antileishmanial activities were evaluated [27,28]. Molecular modeling and dynamic studies were also performed to determine the binding modes and stability of the synthesized compounds in the sterol 14-alpha-demethylase active section.

## 2. Materials and Methods

### 2.1. Chemistry

All chemical agents were provided by the chemical companies of Sigma-Aldrich and Merck. A microwave reactor was used to perform microwave-supported syntheses. (Anton-Paar, Monowave 300, Austria). For the NMR (^1^H-NMR 300 MHz and ^13^C-NMR 75 MHz) and mass spectroscopic analyses, a Fourier transform NMR spectrometer (Bruker Bioscience, Billerica, MA, USA) and a LCMS-IT-TOF system (Shimadzu, Kyoto, Japan) was used, respectively. DMSO-*d_6_* was used as a solvent for NMR applications. TLC applications on silica gel 60 F254 (Merck, Germany) were used to regulate reactions.

*Synthesis of methyl 4-(1H-benz[d]imidazol-2-yl) benzoate (* **1** *)*

The title compound was obtained through a microwave-supported reaction as described previously [29,30,31].

*Synthesis of methyl 4-(1H-benz[d]imidazol-2-yl) benzohydrazide (* **2** *)*

Methyl 4-(1*H*-benz[d]imidazol-2-yl) benzoate (**1**) (0.01 mol) in ethanol (15 mL) and hydrazine hydrate (5 mL) was reacted under microwave irradiation to obtain synthesis of methyl 4-(1*H*-benz[d]imidazol-2-yl) benzohydrazide (**2**), as reported in the literature [29,30,31].

*Synthesis of N-methyl/ethyl-2-[4-(1H-benzimidazol-2-yl)benzoyl]hydrazine-1-carbothioamide (***3a**, **3b***)*

The compound **2** (0.01 mol) and corresponding alkyl isothiocyanate (0.012 mol) in ethanol were refluxed for 2 h. Then, the mixture was cooled, precipitate was filtered, washed with ethanol, and dried [29,30,31].

*Synthesis of 3-(4-(1H-benzimidazol-2-yl)phenyl)-5-mercapto-2-(4-(4-methyl/ethyl)-4H-1,2,4-triazole (***4a**–**4b***)*

The compounds **3a** or **3b** (0.001 mol) in ethanol were refluxed for 2 h in the presence of NaOH (0.012 mol). Then, the solution was acidified with HCl 37%, the precipitated product was filtered, washed with water, dried, and then recrystallized from ethanol [29,30,31].

*Synthesis of target compounds (***5a**–**5h***)*

The compounds **4a** or **4b** (0.001 mol) and a substituted 1-acetyl-2-bromobenzene derivative (0.001 mol) in acetone (10 mL) were reacted in the presence of K_2_CO_3_ to afford target compounds as described previously [29,30,31].

*3-(4-(1H-benzimidazol-2-yl)phenyl)-5-(2-(4-cyanophenyl)-4-methyl-2-oxo-ethylthio)-4H-1,2,4-triazole (***5a***)*: Yield: 82%. ^1^H-NMR δ(ppm): 3.74 (3H, singlet, -CH_3_), 4.99 (2H, singlet, -CH_2_-), 7.37–7.40 (2H, multiplet, BZI C-H), 7.70–7.75 (2H, multiplet, BZI C-H), 7.98 (2H, doublet, *J* = 8.43 Hz, 4-CNPh C-H), 8.05 (2H, doublet, *J* = 8.43, 1,4-DSB C-H), 8.18 (2H, doublet, *J* = 8.49, 4-CNPh C-H), 8.42 (2H, doublet, *J* = 8.34 Hz, 1,4-DSB C-H). ^13^C-NMR δ(ppm): 32.55, 48.43, 115.36, 115.42, 116.02, 118.56, 121.78, 122.17, 123.65, 127.63, 129.34, 129.54, 130.42, 133.31, 139.03, 145.30, 150.58, 154.25, 193.45. [M + H]^+^ calculated for C_25_H_18_N_6_OS: 451.1345; found: 451.1336.

*3-(4-(1H-benzimidazol-2-yl)phenyl)-5-(2-(4-bromophenyl)-4-methyl-2-oxo-ethylthio)-4H-1,2,4-triazole (***5b***)*: Yield: 80%. ^1^H-NMR δ(ppm): 3.72 (3H, singlet, -CH_3_), 4.93 (2H, singlet, -CH_2_-), 7.27–7.32 (2H, multiplet, BZI C-H), 7.66–7.70 (2H, multiplet, BZI C-H), 7.77 (2H, doublet, *J* = 8.55 Hz, 4-BrPh C-H), 7.92–7.99 (4H, multiplet, 1,4-DSB C-H, 4-BrPh C-H), 8.36 (2H, doublet, *J* = 8.40 Hz, 1,4-DSB C-H). ^13^C-NMR δ(ppm): 32.52, 41.09, 113.81, 114.23, 115.58, 123.48, 127.52, 128.40, 128.91, 129.30, 130.71, 130.93, 132.37, 134.77, 138.74, 150.47, 151.05, 155.22, 157.51, 193.21. [M + H]^+^ calculated for C_24_H_18_BrN_6_OS: 504.0471; found: 504.0488.

*3-(4-(1H-benzimidazol-2-yl)phenyl)-5-(2-(4-methylphenyl)-4-methyl-2-oxo-ethylthio)-4H-1,2,4-triazole (***5c***)*: Yield: 79%. ^1^H-NMR δ(ppm): 2.39 (3H, singlet, CH_3_), 3.72 (3H, singlet, -CH_3_), 4.92 (2H, singlet, -CH_2_-), 7.28–7.31 (2H, multiplet, BZI C-H), 7.35–7.38 (2H, multiplet, Ar-C-H), 7.66–7.69 (2H, multiplet, Ar-C-H), 7.92–7.95 (4H, multiplet, Ar-C-H), 8.36 (2H, doublet, *J* = 8.43 Hz, 1,4-DSB C-H). ^13^C-NMR δ(ppm): 21.68, 32.51, 41.24, 113.98, 115.14, 115.55, 123.52, 127.54, 128.99, 129.05, 129.30, 129.84, 130.62, 133.22, 138.68, 139.90, 144.79, 150.44, 151.22, 155.18, 193.36. [M + H]^+^ calculated for C_25_H_21_N_5_OS: 440.1525; found: 440.1540.

*3-(4-(1H-benzimidazol-2-yl)phenyl)-5-(2-(3,4-dihydoxyphenyl)-4-methyl-2-oxo-ethylthio)-4H-1,2,4-triazole (***5d***)*: Yield: 74%. ^1^H-NMR δ(ppm): 3.71 (3H, singlet, -CH_3_), 4.82 (2H, singlet, -CH_2_), 6.85 (1H, doublet, *J* = 8.25 Hz, 3,4-DiOHPh C-H), 7.25 (1H, double doublet, *J* = 8.56–3.12 Hz, BZI C-H), 7.39–7.46 (2H, multiplet, Ar-C-H), 7.63–7.66 (2H, multiplet, Ar-CH), 7.91 (2H, doublet, *J* = 8.40 Hz, 1,4-DSB C-H), 8.34 (2H, doublet, *J* = 8.37 Hz, 1,4-DSB C-H), 9.46 (1H, singlet, -OH), 10.06 (1H, singlet, -OH). ^13^C-NMR δ(ppm): 32.45, 41.07, 115.08, 115.64, 116.34, 122.52, 123.03, 124.45, 127.31, 127.51, 128.62, 128.87, 129.26, 129.42, 131.52, 138.10, 145.83, 150.75, 151.32, 151.86, 155.23, 191.89. [M + H]^+^ calculated for C_24_H_19_N_5_O_3_S: 458.1280; found: 458.1281.

*3-(4-(1H-benzimidazol-2-yl)phenyl)-5-(2-(4-cyanophenyl)-4-ethyl-2-oxo-ethylthio)-4H-1,2,4-triazole (***5e***)*: Yield: 82%. ^1^H-NMR δ(ppm): 1.28 (3H, t, *J* = 7.08, -CH_3_), 4.12 (2H, q, *J* = 7.02 Hz, -CH_2_), 5.04 (2H, singlet, -CH_2_), 7.24 (1H, double doublet, *J* = 8.57–1.94 Hz, BZI C-H),7.62–7.65 (2H, multiplet, BZI C-H), 7.83 (2H, doublet, *J* = 8.34 Hz, 4-CNPh C-H), 8.05 (2H, doublet, *J* = 8.31, 1,4-DSB C-H), 8.19 (2H, doublet, *J* = 8.34, 4-CNPh C-H), 8.34 (2H, doublet, *J* = 8.31 Hz, 1,4-DSB C-H), 13.08 (1H, singlet, BZI-NH). ^13^C-NMR δ(ppm): 15.52, 36.57, 41.15, 111.98, 116.04, 118.56, 119.54, 122.54, 123.24, 127.39, 128.57, 129.04, 129.27, 129.52, 131.96, 133.30, 139.05, 150.33, 150.78, 154.86, 193.34. [M + H]^+^ calculated for C_26_H_20_N_6_OS: 465.1482; found: 465.1492.

*3-(4-(1H-benzimidazol-2-yl)phenyl)-5-(2-(4-bromophenyl)-4-ethyl-2-oxo-ethylthio)-4H-1,2,4-triazole (***5f***)*: Yield: 74%. ^1^H-NMR δ(ppm): 1.28 (3H, t, *J* = 7.14, -CH_3_), 4.12 (2H, q, *J* = 7.20 Hz, -CH_2_), 5.01 (2H, singlet, -CH_2_-), 7.23–7.26 (2H, multiplet, BZI C-H), 7.38–7.44 (2H, multiplet, BZI C-H), 7.84 (2H, doublet, *J* = 8.46, 4-BrPh), 8.12–8.17 (4H, multiplet, 1,4-DSB C-H, 4-BrPh), 8.34 (2H, doublet, *J* = 8.43 Hz, 1,4-DSB C-H), 13.08 (1H, singlet, BZI -NH). ^13^C-NMR δ(ppm): 15.53, 35.12, 41.10, 111.97, 116.23, 116.52, 119.56, 122.38, 123.38, 127.38, 128.62, 129.28, 131.95, 132.08, 132.59, 135.55, 144.29, 150.51, 150.79, 154.80, 192.40. [M + H]^+^ calculated for C_25_H_20_BrN_5_OS: 518.0646; found: 518.0645.

*3-(4-(1H-benzimidazol-2-yl)phenyl)-5-(2-(4-methylphenyl)-4-ethyl-2-oxo-ethylthio)-4H-1,2,4-triazole (**5g**)*: Yield: 74%. ^1^H-NMR δ(ppm): 1.28 (3H, t, *J* = 7.11, -CH_3_), 2.40 (3H, singlet, -CH_3_), 4.11 (2H, q, *J* = 7.20 Hz, -CH_2_), 4.98 (2H, singlet, -CH_2_-), 7.21–7.26 (2H, multiplet, Ar C-H), 7.37 (2H, doublet, *J* = 8.04, 4-CH_3_Ph C-H), 7.62–7.66 (2H, multiplet, BZI C-H), 7.83 (2H, doublet, *J* = 8.34 Hz, 1,4-DSB C-H), 7.95 (2H, doublet, *J* = 8.13, 4-CH_3_Ph C-H), 8.34 (2H, doublet, *J* = 8.34 Hz, 1,4-DSB C-H), 13.08 (1H, singlet, BZI-NH). ^13^C-NMR δ(ppm):15.55, 21.69, 35.82, 41.22, 111.29, 114.41, 118.16, 122.90, 127.38, 128.65, 129.03, 129.28, 129.85, 131.07, 131.93, 132.74, 133.27, 144.79, 150.60, 150.80, 154.77, 193.22. [M + H]^+^ calculated for C_26_H_23_N_5_OS: 454.1683; found: 454.1696.

*3-(4-(1H-benzimidazol-2-yl)phenyl)-5-(2-(3,4-dihydroxyphenyl)-4-ethyl-2-oxo-ethylthio)-4H-1,2,4-triazole (***5h***)*: Yield: 78%. ^1^H-NMR δ(ppm): 1.27 (3H, t, *J* = 7.11, -CH_3_), 4.11 (2H, q, *J* = 7.17 Hz, -CH_2_), 4.88 (2H, singlet, -CH_2_-), 6.85 (1H, doublet, *J* = 8.28 Hz, 3,4-DiOHPh C-H), 7.25 (1H, double doublet, *J* = 8.54–1.85 Hz, BZI C-H), 7.40–7.48 (2H, multiplet, 3,4-DiOHPh C-H), 7.59–7.68 (2H, multiplet, BZI C-H), 7.83 (2H, doublet, *J* = 8.46 Hz, 1,4-DSB C-H), 8.34 (2H, doublet, *J* = 8.43 Hz, 1,4-DSB C-H), 9.45 (1H, singlet, -OH), 10.04 (1H, singlet, -OH), 13.08 (1H, singlet, BZI -NH). ^13^C-NMR δ(ppm): 15.55, 36.24, 41.08, 111.28, 115.66, 118.85, 119.64, 122.48, 123.25, 124.58, 127.38, 127.55, 128.69, 129.29, 131.90, 133.15, 133.72, 145.83, 150.79, 151.85, 152.41, 154.73, 191.74. [M + H]^+^ calculated for C_25_H_21_N_5_O_3_S: 472.1436; found: 472.1438.

### 2.2. Culture of the Leishmania tropica Isolate

The parasite isolates were obtained from the parasite bank of the Department of Parasitology, Faculty of Medicine of Manisa Celal Bayar University. The preparation of the promastigotes obtained is as follows.

*Leishmania tropica* isolate, which was coded as MHOM/TR/2012/CBU17, was used in the study. The isolate, stored in liquid nitrogen, was thawed by keeping it in a 37 °C water bath for two minutes after being removed from the storage environment; after the thawing process, NNN media were inoculated, and the media were incubated in an oven at 25 °C. The reproductive status of the parasites was checked by preparing slides and coverslips every other day. After parasite reproduction took place, parasites were inoculated from NNN medium to RPMI-1640 (Gibco^TM^, Paisley, Scotland, UK) medium to obtain more parasites. Prior to its use, 10% FCS, 1% penicillin/streptomycin, and 1% gentamicin were added to the commercially obtained medium. Distribution was made with 5 milliliters per 25 mL flash, and cultivation was performed by adding 50 μL of the reproduced promastigotes. Flasks used for cultivation were incubated on a 25 °C stove. The reproduction of the parasites was monitored, and the medium was added every 2–3 days. Afterward, the number of promastigotes was counted using the Thoma lam as 10^6^ promastigotes/mL, and a suspension containing promastigotes was prepared [32].

The synthesized compounds were prepared at their various 12 concentrations through serial dilutions (0.48 µg/mL–1000 µg/mL), using 2% DMSO solution to evaluate their capacity to prevent the development of the *Leishmania tropica* isolate. The 100 µL of each compound concentration was added to 96-well plates in triplicate. Then, the prepared solution of promastigotes was added to each well of 100 µL. All well plates were left to incubate at 25 °C for 48 h. Following the incubation period, in order to measure the absorbance, the XTT (Biotium, Fremont, CA, USA) (sodium 3,39-[1-(phenylaminocarbonyl)-3,4-tetrazolium]-bis(4-methoxy-6-nitro) benzene sulphonic acid monohydrate) cell viability assay kit was prepared, and a 50 µL aliquot of this solution was added to each well of the plates. In order to calculate the viability percentage of the compounds that were considered to be effective according to the XTT method, the substances were diluted from a high dose to low dose in the study. Serial dilutions were repeated three times on the same plate and at different times to be able to increase the reliability of the viability calculation. The absorbance was recorded at 450 nm after 4 h incubation at 37 °C. The viability percentages of the test compounds were calculated according to the following Equation [33,34]:

$$\%Viability=\frac{\left(A\left(S\right)-A\left(B\right)\right)}{\left(A\left(C\right)-A\left(B\right)\right)}*100$$

*B*: blank, *C*: control, *A*(*B*): the absorbance of blank, *A*(*C*): the absorbance of control, *A*(*S*): the absorbance of sample.

### 2.3. Molecular Docking

Molecular docking studies were carried out using PDB ID:3L4D crystal. This crystal belongs to the 14-alpha-demethylase (CYP51) enzyme from Leishmania [35]. The relevant X-ray crystal was obtained using Protein Data Bank server (www.pdb.org, accessed on 1 June 2023). Schrödinger Maestro [36] interface, LigPrep module [37], and Glide module [38] were used throughout the docking procedure. Standard precision (SP) docking mode was used.

### 2.4. Molecular Dynamic Simulations

Molecular dynamic studies to check the stability of the compound were carried out for 100 ns [39]. Desmond application [40] was used for molecular dynamics studies. System setup was achieved using the POPE membrane model, OPLS3e force field, and TIP3P water model [41]. Stability parameters (Rg, RMSF, RMSD) were calculated with the Desmond application [42].

## 3. Results

### 3.1. Chemistry

**Scheme 1:**
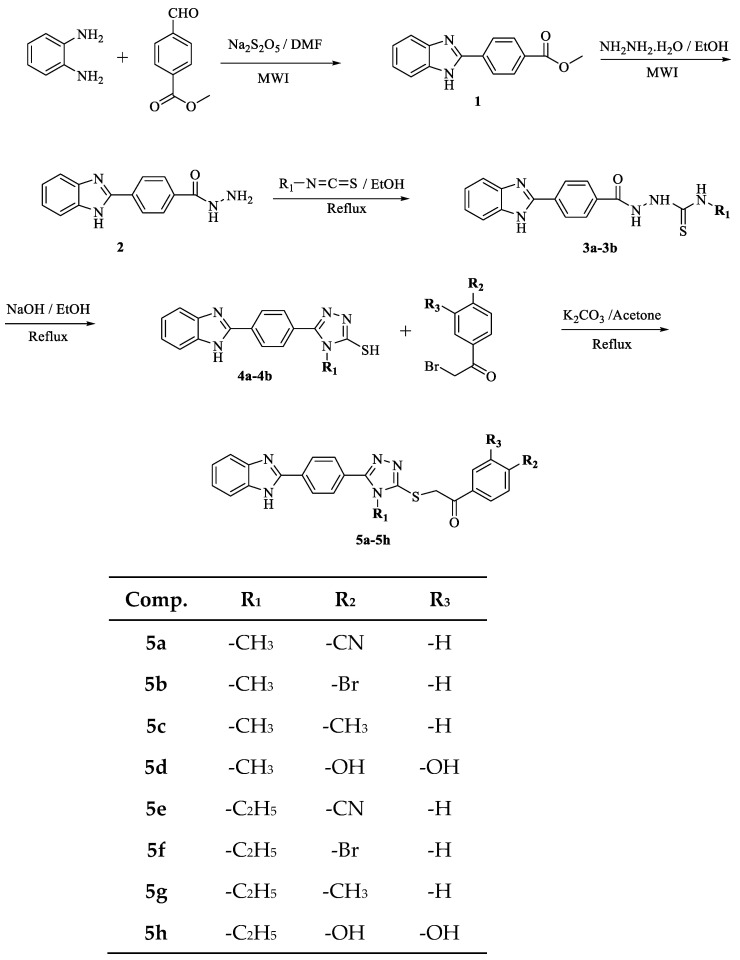
Synthesis route and substituents for final compounds.

The syntheses of the final compounds (**5a**–**5h**) were performed in five steps as outlined in Scheme 1. First, the treatment of methyl 4-formylbenzoate with o-phenylenediamines in the presence of Na_2_S_2_O_5_ resulted in the isolation of the desired product **1**. Secondly, hydrazide derivative **2** was developed with compound **1** and an excess of hydrazine hydrate. Thirdly, to obtain thioureas (**3a**–**3b**), hydrazide derivative **2** and the substituted isothiocyanate were reacted. Compounds **4a**–**4b** were then synthesized using NaOH by means of a ring closure reaction. In the last step, the S-alkylation reaction of **4a** or **4b** with a 2-bromoacetophenone in the presence of potassium carbonate gave the final derivatives (**5a**–**5h**). The structure of the synthesized compounds was confirmed by spectral data. The protons of the final compounds were observed at expected areas in ^1^H NMR spectra. Similarly, all carbon signals in ^13^C NMR spectra are in an agreement with the theorical peak values. HRMS spectra confirmed the calculated molecular weight of the entire compounds.

### 3.2. Antileishmanial Activity

Table 1 presents the % viability values of the parasites against the synthesized compounds and reference drug amphotericin B. As seen from this table, compounds **5b**, **5c,** and **5g** displayed no efficiency against *Leishmania tropica*; however, it was detected that compounds **5a**, **5d**, **5e**, **5f**, and **5h** showed antileishmanial activity at various rates. Among these derivatives, compound **5f** displayed a good activity, because the percentage viability of *Leishmania tropica* was determined as 20% at a concentration of 1000 µg/mL. It was determined that compound **5f** could inhibit the growth of *Leishmania tropica* by 80%. Moreover, it was observed that compound **5h** exhibited antileishmanial activity at a similar level to compound **5f**. The percentage viability of the promastigotes with this compound was calculated as 29%. The IC_50_ values for the compounds **5d**, **5f**, and **5h** were calculated as 239.03 μg/mL, 460.37 μg/mL and 468.01 μg/mL, respectively. The percentage viability of the promastigotes for the other three effective derivatives, compounds **5a**, **5d**, and **5e**, were determined as 72%, 30%, and 82%, respectively.

### 3.3. Molecular Docking

**Figure 1:**
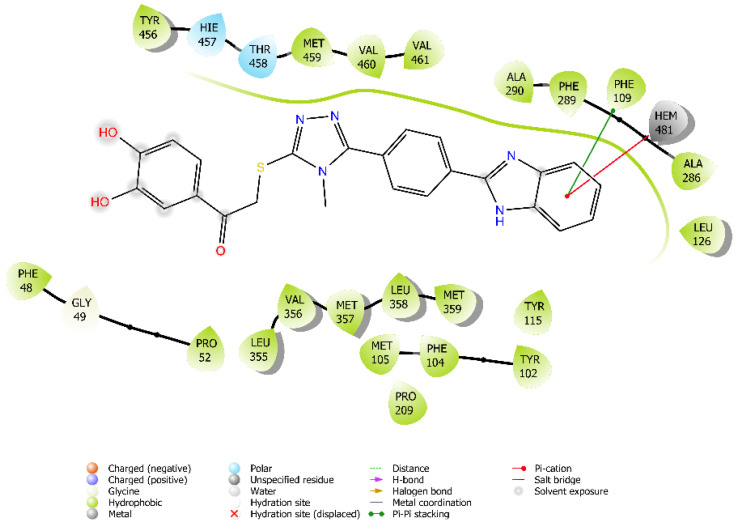
Two-dimensional interaction pose of compound **5d** and 14-alpha-demethylase (CYP51) (PDB ID:3L4D).

**Figure 2:**
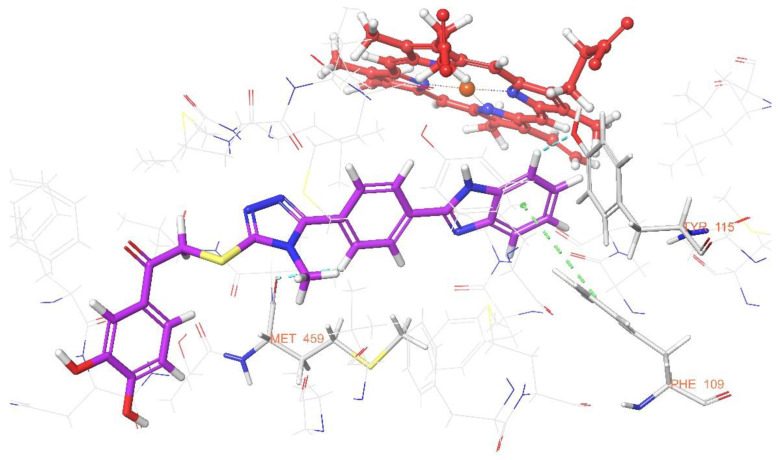
Three-dimensional interaction pose of compound **5d** and 14-alpha-demethylase (CYP51) (PDB ID:3L4D).

**Figure 3:**
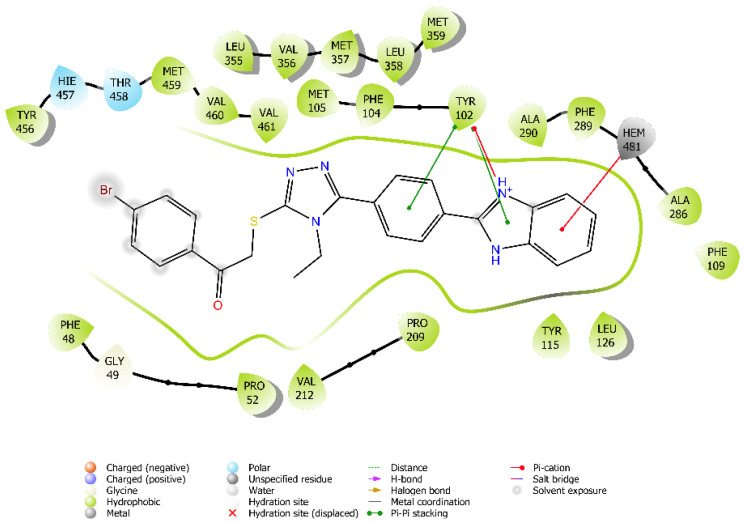
Two-dimensional interaction pose of compound **5f** and 14-alpha-demethylase (CYP51) (PDB ID:3L4D).

**Figure 4:**
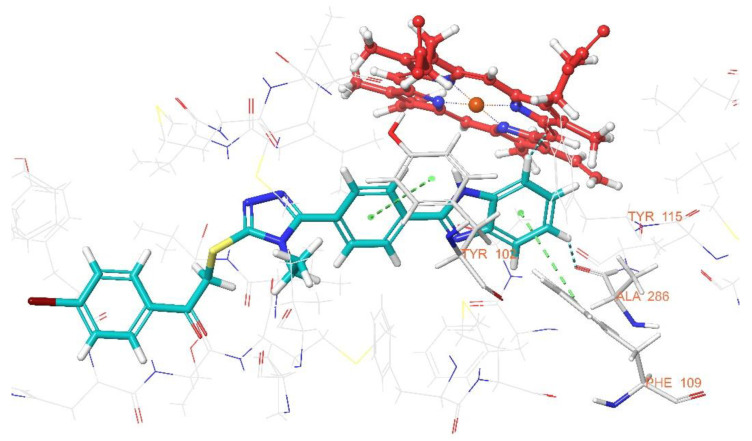
Three-dimensional interaction pose of compound **5f** and 14-alpha-demethylase (CYP51) (PDB ID:3L4D).

**Figure 5:**
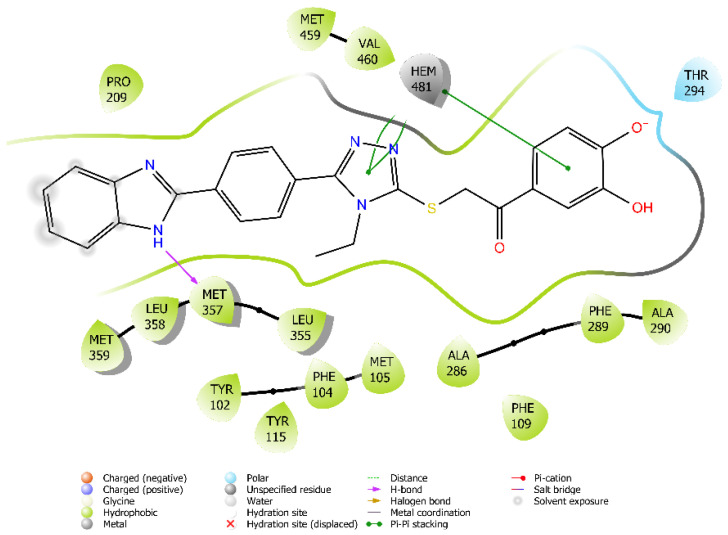
Two-dimensional interaction pose of compound **5h** and 14-alpha-demethylase (CYP51) (PDB ID:3L4D).

**Figure 6:**
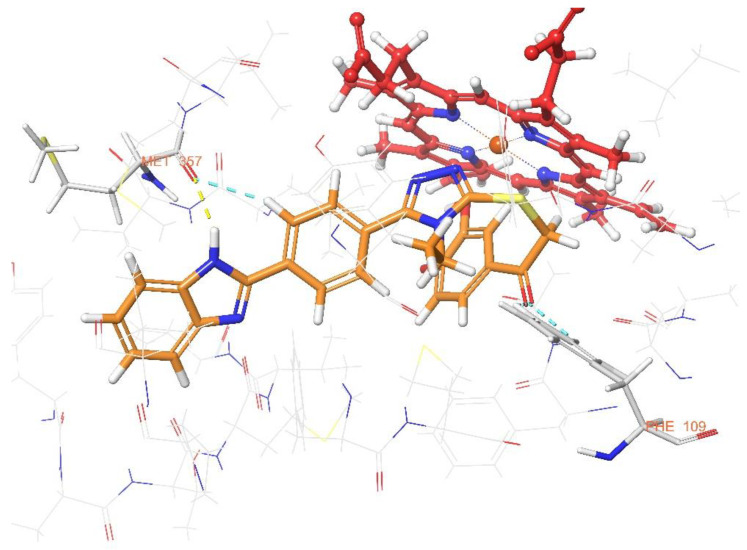
Three-dimensional interaction pose of compound **5h** and 14-alpha-demethylase (CYP51) (PDB ID:3L4D).

Docking poses performed with the PDB ID:3L4D enzyme for compounds **5d**, **5f**, and **5h** are presented in Figure 1, Figure 2, Figure 3, Figure 4, Figure 5 and Figure 6. The poses for compound **5d** are shown in Figure 1 and Figure 2. These positions demonstrate the cation-pi interaction between Hem481 and molecule 5d’s benzimidazole ring. The benzimidazole ring also shows pi-pi interaction with the phenyl of Phe109. Finally, benzimidazole forms aromatic hydrogen bonds with the hydroxy group of Tyr115.

The poses for compound **5f** are shown in Figure 3 and Figure 4. According to these poses, cation-pi interaction is observed between the benzimidazole ring of compound **5f** and Hem481. The benzimidazole ring also shows pi-pi interaction with the phenyl of Phe109. The phenyl ring of compound **5f** shows pi-pi interaction with the phenyl ring of Tyr102. Finally, benzimidazole ring forms aromatic hydrogen bonds with the carbonyl group of Ala286.

The poses for compound **5h** are shown in Figure 5 and Figure 6. According to these poses, three pi-pi interactions are observed between the phenyl and triazole rings of compound **5f** and Hem481. Additionally, the nitrogen of the benzimidazole ring forms a hydrogen bond with the carbonyl group of Met357. The phenyl ring of compound **5h** forms an aromatic hydrogen bond with the carbonyl group of Met357. Finally, the carbonyl group of compound **5h** forms an aromatic hydrogen bond with the phenyl ring of Phe109.

Another striking point is that compound **5h** is placed in the opposite position to the enzyme. Compared with compound **5f**, compound **5f** contains a 4-bromophenyl ring, while compound **5h** contains a 3,4-dihydroxyphenyl ring. This substituent difference may have affected the conformation and placement of compound **5h**. Another derivative containing a 3,4-dihydroxyphenyl ring, compound **5d**, is positioned to interact with the benzimidazole ring HEM481 (Figure 1). The difference between compound **5d** and compound **5h** is the substituents that they carry on the triazole ring. While compound **5d** carries a methyl chain, compound **5h** carries an ethyl chain. The introduction of the ethyl group affected the conformation of the compound and its placement on the enzyme active site.

The poses of compounds **5c** and **5g**, which are inactive compounds, are presented in the supporting information file (Figures S1 and S2). The fact that neither compound interacts with HEM481 shows that the activity results are in agreement with in silico studies.

### 3.4. Molecular Dynamic Simulations

**Figure 7:**
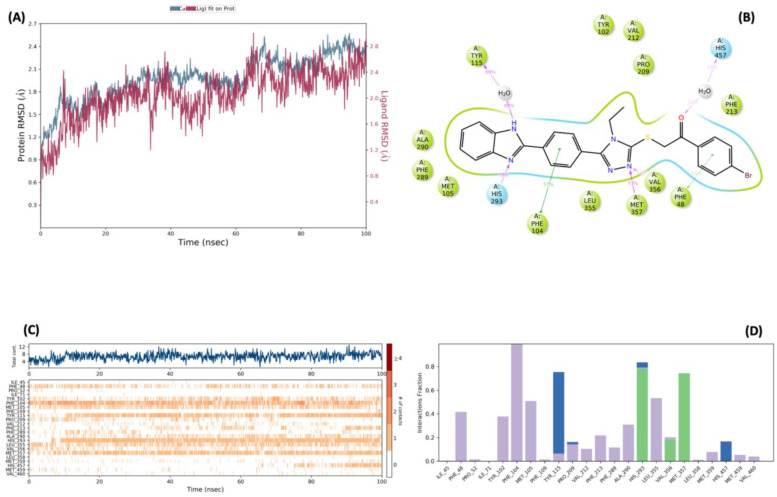
The stability results of compound **5f**. (**A**). RMSD (Protein RMSD is shown in grey while RMSD of compound 5f are shown in red) (**B**). 2D Interaction diagram (cut off 10%) (**C**). Protein–ligand contact analysis (**D**). Amino acid interaction histogram of MD trajectory.

Dynamic studies were carried out on the **5f** + 3L4D complex for 100 ns. The purpose of molecular dynamics studies is to measure the stability of the complex formed by the ligand with the enzyme’s active site. The parameter used for this is the RMSD parameter. It can be seen that the RMSD value presented in Figure 7A is a maximum of 2.7 Å. The fact that this value is between 1 and 3 Å indicates the stability of the complex. Therefore, the stability of the compound (**5f**) in the enzyme active site has been demonstrated.

Figure 7B presents two-dimensional interactions of the compound **5f** + 3L4D complex. Amino acids containing 10% or more interactions have been demonstrated. Of these interactions, Tyr115, His293, Phe104, and Met357 in particular exhibited a more than 50% interaction. In Figure 7C, the interaction residues with amino acids are visible for 100 ns. Here, an uninterrupted interaction is seen, especially with the amino acids Phe104, Tyr115, His293, and Met357. The contribution of these amino acids to stability is understood once again. Figure 7D presents the interactions as a bar graph.

The molecular dynamics studies also show the interaction of the benzimidazole ring with the iron in the middle of HEM481 at 3–10 ns. This interaction, which started at the time of writing, continues uninterruptedly.

In addition to these interactions, the aromatic hydrogen bonds made by compound **5f** are seen when the video is watched. The monosubstituted phenyl ring forms an aromatic hydrogen bond with Met357, Met459, and Tyr102. Aromatic hydrogen bonds are formed between Tyr456, His457, Thr458, and Pro209 and the 1,4-disubstituted phenyl ring. The benzimidazole ring forms aromatic hydrogen bonds with Ala286 and Tyr115. The carbonyl group forms aromatic hydrogen bonds with Phe213 and Phe48.

## 4. Discussion

In this study, the in vitro antileishmanial activity of eight new benzimidazole-triazole derivatives was investigated. The XTT-based methodology used for this purpose is a reliable and effective way to evaluate anti-leishmanial properties in primitive biological materials and developed chemical compounds.

The target compounds were synthesized using conventional chemical methods. The structures of the obtained compounds were confirmed through spectroscopic analyses.

According to the biological activity results, among the synthesized compounds, five compounds (**5a**, **5d**, **5e**, **5f**, and **5h**) showed antileishmanial activity at various rates. On the other hand, the remaining three compounds in the series displayed no efficiency. Compounds **5d**, **5f**, and **5h**, which are the most active compounds, were subjected to molecular docking studies. It seems that the activity studies and in silico studies are in harmony in terms of their placement and interaction on the enzyme’s active site. For compounds **5d** and **5f**, the interaction of the benzimidazole ring with HEM481 demonstrates the importance of the benzimidazole ring for activity. Compound **5f,** which was selected for dynamics studies, maintained its interactions and stability for 100 ns and once again demonstrated its activity potential.

Although many drug combinations have been developed in therapy, new drug development is needed due to the emergence of resistance. Within the scope of this study, which we conducted for this purpose, it was observed that five of the new compounds whose antileishmanial activity was investigated stopped the growth of *Leishmania tropica* promastigotes at different degrees. Additionally, molecular docking and dynamic investigations emphasized the biological significance of compounds **5d**, **5f**, and **5h**.

## 5. Conclusions

In conclusion, the results of the current study displayed the potential of synthetic benzimidazole-triazole against *Leishmania tropica*. In the light of these results, we believe that the compounds showing antileishmanial activity can be antileishmanial drug candidates after their safety and efficacy have been confirmed through more in vitro and in vivo studies. Based on their chemical structures, further modification could increase their activity. Their selectivity against mammal cells should then be assayed versus parasites. Furthermore, the in vitro activity against sterol 14-alpha-demethylase might be tested.

## Figures and Tables

**Table 1:** % viability of parasites determined colorimetrically via XTT method after 48 h incubation (10^6^ promastigote/mL) and calculated IC_50_ (μg/mL) values (N.c: not calculated).

Compound	Concentration (μg/mL)												
	1000	500	250	125	62.50	31.25	15.63	7.81	3.91	1.95	0.98	0.49	IC_50_
**5a**	72	78	100	100	100	100	100	100	100	100	100	100	N.c
**5b**	100	100	100	100	100	100	100	100	100	100	100	100	N.c
**5c**	100	100	100	100	100	100	100	100	100	100	100	100	N.c
**5d**	30	41	61	100	100	100	100	100	100	100	100	100	239.03
**5e**	82	100	100	100	100	100	100	100	100	100	100	100	N.c
**5f**	20	35	100	100	100	100	100	100	100	100	100	100	460.37
**5g**	100	100	100	100	100	100	100	100	100	100	100	100	N.c
**5h**	29	46	100	100	100	100	100	100	100	100	100	100	468.01
Drug Control (Amphotericin B)	0	0	0	0	0	0	0	0	0	0	0	0	<0.49
Parasite Control	100	100	100	100	100	100	100	100	100	100	100	100	N.c

## Data Availability

The data of this study are available upon request from the corresponding author.

## Supplementary Materials

The following supporting information can be downloaded at: https://www.mdpi.com/article/10.3390/vetsci10110648/s1, Figures S1–S25: Spectroscopic spectra of the compounds **5a**–**5h**; Video S1: Molecular dynamics studies visualization of compound **5h**.

## References

[1] World Health Organization (WHO) Leishmaniasis. https://www.who.int/news-room/fact-sheets/detail/leishmaniasis.

[2] Murray H.W., Berman J.D., Davies C.R., Saravia N.G. (2005). Advances in *Leishmaniasis*. Lancet.

[3] Chernin J. (2000). Parasitology.

[4] Rohmer M., Bouvier P., Ourisson G. (1979). Molecular Evolution of Biomembranes: Structural Equivalents and Phylogenetic Precursors of Sterols. Proc. Natl. Acad. Sci. USA.

[5] Volkman J.K. (2005). Sterols and other Triterpenoids: Source Specificity and Evolution of Biosynthetic Pathways. Org. Geochem..

[6] Nes W.R., McKean M.L. (1977). Biochemistry of Steroids and Other Isoprenoids.

[7] Lepesheva G.I., Waterman M.R. (2007). Sterol 14-alpha-demethylase Cytochrome P450 (CYP51), a P450 in all Biological Kingdoms. Biochim. Biophys. Acta.

[8] Edwards P.A., Ericsson J. (1999). Sterols and Isoprenoids: Signaling Molecules Derived from the Cholesterol Biosynthetic Pathway. Annu. Rev. Biochem..

[9] Roberts C.W., McLeodm R., Rice D.W., Ginger M., Chance M.L., Goad L.J. (2003). Fatty Acid and Sterol Metabolism: Potential Antimicrobial Targets in Apicomplexan and Trypanosomatid Parasitic Protozoa. Mol. Biochem. Parasitol..

[10] Zhou W., Cross G.A., Nes W.D. (2007). Cholesterol Import Fails to Prevent Catalyst-Based Inhibition of Ergosterol Synthesis and Cell Proliferation of *Trypanosoma brucei*. J. Lipid Res..

[11] Lepesheva G.I., Park H.W., Hargrove T.Y., Vanhollebeke B., Wawrzak Z., Harp J.M., Sundaramoorthy M., Nes W.D., Pays E., Chaudhuri M. (2010). Crystal Structures of *Trypanosoma brucei* Sterol 14alpha-demethylase and Implications for Selective Treatment of Human Infections. J. Biol. Chem..

[12] Yao C., Wilson M.E. (2016). Dynamics of Sterol Synthesis during Development of *Leishmania* spp. Parasites to Their Virulent form. Parasit. Vectors.

[13] Xu W., Hsu F.F., Baykal E., Huang J., Zhang K. (2014). Sterol Biosynthesis is Required for Heat Resistance but not Extracellular Survival in Leishmania. PLoS Pathog..

[14] Petrikkos G., Skiada A. (2007). Recent Advances in Antifungal Chemotherapy. Int. J. Antimicrob. Agents.

[15] Zonios D.I., Bennet J.E. (2008). Update on Azole Antifungals. Semin. Respir. Crit. Care Med..

[16] Docampo R., Moreno S.N., Turrens J.F., Katzin A.M., Gonzalez-Cappa S.M., Stoppani A.O. (1981). Biochemical and Ultrastructural Alterations Produced by Miconazole and Econazole in *Trypanosoma cruzi*. Mol. Biochem. Parasitol..

[17] Beach D.H., Goad L.J., Holz G.G. (1986). Effects of Ketoconazole on Sterol Biosynthesis by *Trypanosoma cruzi* Epimastigotes. Biochem. Biophys. Res. Commun..

[18] Urbina J.A., Lazardi K., Aguirre T., Piras M.M., Piras R. (1988). Antiproliferative Synergism of the Allylamine SF 86–327 and Ketoconazole on Epimastigotes and Amastigotes of *Trypanosoma (Schizotrypanum) cruzi*. Antimicrob. Agents Chemother..

[19] Apt W., Aguilera X., Arribada A., Pérez C., Miranda C., Sánchez G., Zulantay I., Cortés P., Rodriguez J., Juri D. (1998). Treatment of Chronic Chagas’ Disease with Itraconazole and Allopurinol. Am. J. Trop. Med. Hyg..

[20] Araújo M.S., Martins-Filho O.A., Pereira M.E., Brener Z.J. (2000). A Combination of Benzimidazole and Ketoconazole Enhances Efficacy of Chemotherapy of Experimental Chagas’ disease. Antimicrob. Chemother..

[21] Molina J., Martins-Filho O., Brener Z., Romanha A.J., Loebenberg D., Urbina J.A. (2000). Activities of the Triazole Derivative SCH 56592 (posaconazole) Against Drug-Resistant Strains of the Protozoan Parasite *Trypanosoma (Schizotrypanum) cruzi* in Immunocompetent and Immunosuppressed Murine Hosts. Antimicrob. Agents Chemother..

[22] Buckner F.S. (2008). Sterol 14-demethylase Inhibitors for *Trypanosoma cruzi* Infections. Adv. Exp. Med. Biol..

[23] Lepesheva G.I., Ott R.D., Hargrove T.Y., Kleshchenko Y.Y., Schuster I., Nes W.D., Hill G.C., Villalta F., Waterman M.R. (2007). Sterol 14alpha-demethylase as a Potential Target for Antitrypanosomal Therapy: Enzyme Inhibition and Parasite Cell Growth. Chem. Biol..

[24] Urbina J.A., Payares G., Sanoja C., Molina J., Lira R., Brener Z., Romanha A.J. (2003). Parasitological Cure of Acute and Chronic Experimental Chagas disease Using the Long-Acting Experimental Triazole TAK-187. Activity Against Drug-Resistant *Trypanosoma cruzi* Strains. Int. J. Antimicrob. Agents.

[25] Urbina J.A., Payares G., Contreras L.M., Liendo A., Sanoja C., Molina J., Piras M., Piras R., Perez N., Wincker P. (1998). Antiproliferative Effects and Mechanism of Action of SCH 56592 against *Trypanosoma (Schizotrypanum) cruzi*: In Vitro and In Vivo Studies. Antimicrob. Agents Chemother..

[26] Lepesheva G.I., Hargrove T.Y., Kleshchenko Y., Nes W.D., Villalta F., Waterman M.R. (2008). CYP51: A Major Drug Target in the Cytochrome P450 Superfamily. Lipids.

[27] Keurulainen L., Siiskonen A., Nasereddin A., Kopelyanskiy D., Sacerdoti-Sierra N., Leino T.O., Kiuru P. (2015). Synthesis and Biological Evaluation of 2-arylbenzimidazoles Targeting *Leishmania donovani*. Bioorg. Med. Chem. Lett..

[28] Zeyrek F.Y., Gürses G., Uluca N., Doni N.Y., Toprak S., Yesilova Y., Çulha G. (2014). Is the Agent of Cutaneous Leishmaniasis in Sanliurfa changing? First Cases of *Leishmania majör*. Turk. Parazitol. Derg..

[29] Acar Cevik U., Saglik B.N., Levent S., Osmaniye D., Kaya Cavuşoglu B., Ozkay Y., Kaplancikli Z.A. (2019). Synthesis and AChE-inhibitory Activity of New Benzimidazole Derivatives. Molecules.

[30] Can N.Ö., Acar Çevik U., Sağlık B.N., Levent S., Korkut B., Özkay Y., Koparal A.S. (2017). Synthesis, Molecular Docking Studies and antifungal activity evaluation of New Benzimidazole-Triazole as Potential Lanosterol 14α-Demethylase Inhibitors. J. Chem..

[31] Karaca Gençer H., Acar Çevik U., Levent S., Sağlık B.N., Korkut B., Özkay Y., Ilgın S., Öztürk Y. (2017). New Benzimidazole-1,2,4-Triazole Hybrid Compounds: Synthesis, Anticandidal Activity and Cytotoxicity Evaluation. Molecules.

[32] Özbilgin A., Çavuş İ., Yıldırım A., Kaya T., Ertabaklar H. (2018). Evaluation of In vitro and In vivo Drug Efficacy Over *Leishmania tropica*: A Pilot Study. Turk. Parazitol. Derg..

[33] Williams C., Espinosa O.A., Montenegro H., Cubilla L., Capson T.L., Ortega-Barrıa E., Romero L.I. (2003). Hydrosoluble formazan XTT: Its application to natural products drug discovery for Leishmania. J. Microbiol. Methods.

[34] Özbilgin A., Zeyrek F.Y., Güray M.Z., Çulha G., Akyar I., Harman M., Gündüz C. (2020). Determination of antimony resistance mechanism of *Leishmania tropica* causing cutaneous leishmaniasis in Turkey. Mikrobiyoloji Bul..

[35] Hargrove T.Y., Friggeri L., Wawrzak Z., Qi A., Hoekstra W.J., Schotzinger R.J., Lepesheva G.I. (2017). Structural Analyses of Candida Albicans Sterol 14α-Demethylase Complexed with Azole Drugs Address the Molecular Basis of Azole-Mediated Inhibition of Fungal Sterol Biosynthesis. J. Biol. Chem..

[36] (2020). Maestro.

[37] Schrödinger (2020). LigPrep.

[38] Schrödinger (2020). Glide.

[39] Liu X., Shi D., Zhou S., Liu H., Liu H., Yao X. (2018). Molecular Dynamics Simulations and Novel Drug Discovery. Expert Opin. Drug Discov..

[40] M.D.I. Tools (2020). Schrödinger Release 2018-3: Prime, 2018.

[41] Saral A., Sudha P., Muthu S., Sevvanthi S., Sangeetha P., Selvakumari S. (2021). Vibrational Spectroscopy, Quantum Computational and Molecular Docking Studies on 2-Chloroquinoline-3-Carboxaldehyde. Heliyon.

[42] Schrödinger Release (2014). 1: Desmond Molecular Dynamics System.

