# Supplementary material for: In Vitro and In Silico Evaluations of the Antileishmanial Activities of New Benzimidazole-Triazole Derivatives
Source: Vet Sci. 2023 Nov 9;10(11):648. doi: 10.3390/vetsci10110648 (PMC10675599; doi:10.3390/vetsci10110648)
Supplement: Supplementary file 1 [file vetsci-10-00648-s001.zip › vetsci-2670140-Figures S1-S25.pdf]

In Vitro and In Silico Evaluations of the Antileishmanial Activities of New Benzimidazole-Triazole Derivatives

Mustafa Eser [1,*] and ˙Ibrahim Çavu¸s [2]

1 Health Programs, Faculty of Open Education, Anadolu University, Eskisehir 26470, Turkey
2 Department of Parasitology, Faculty of Medicine, Manisa Celal Bayar University, Manisa 45030, Turkey; ibrahim.cavus@cbu.edu.tr
* Correspondence: meser961@anadolu.edu.tr; Tel.: +90-222-3350580 (ext. 3793); Fax: +90-222-3350750

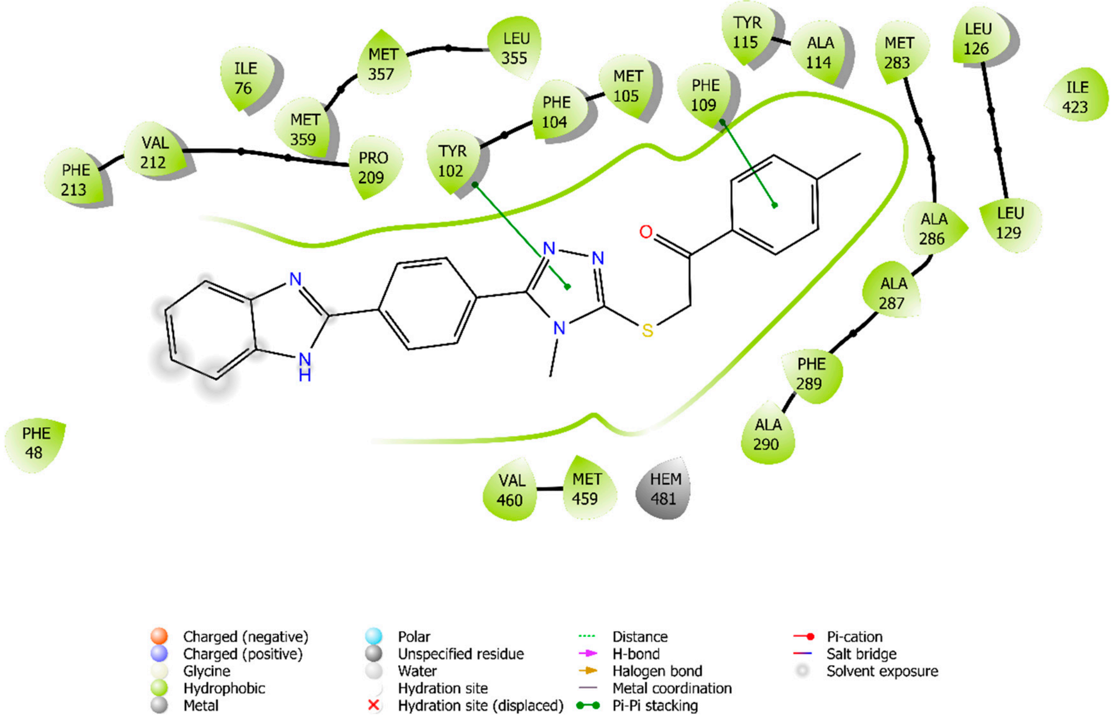

Figure S1. 2D interaction pose of compound **5c** and 14-alpha demethylase (CYP51) (PDB ID:3L4D).

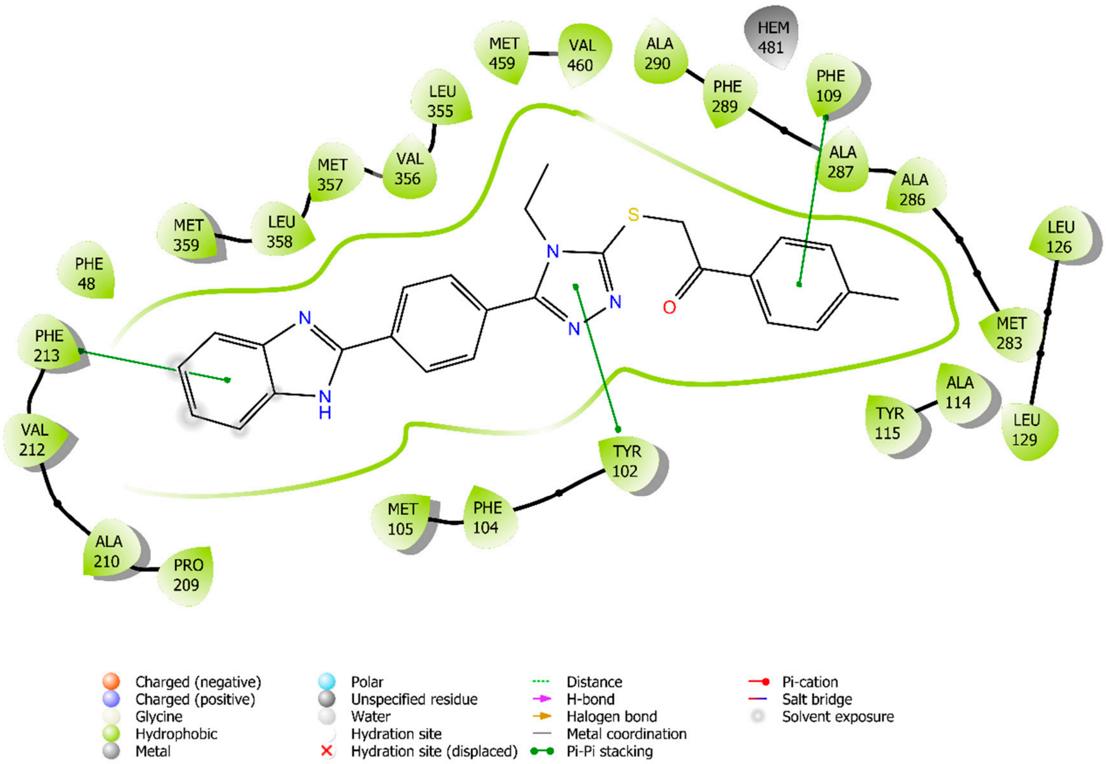

Figure S2. 2D interaction pose of compound **5g** and 14-alpha demethylase (CYP51) (PDB ID:3L4D).

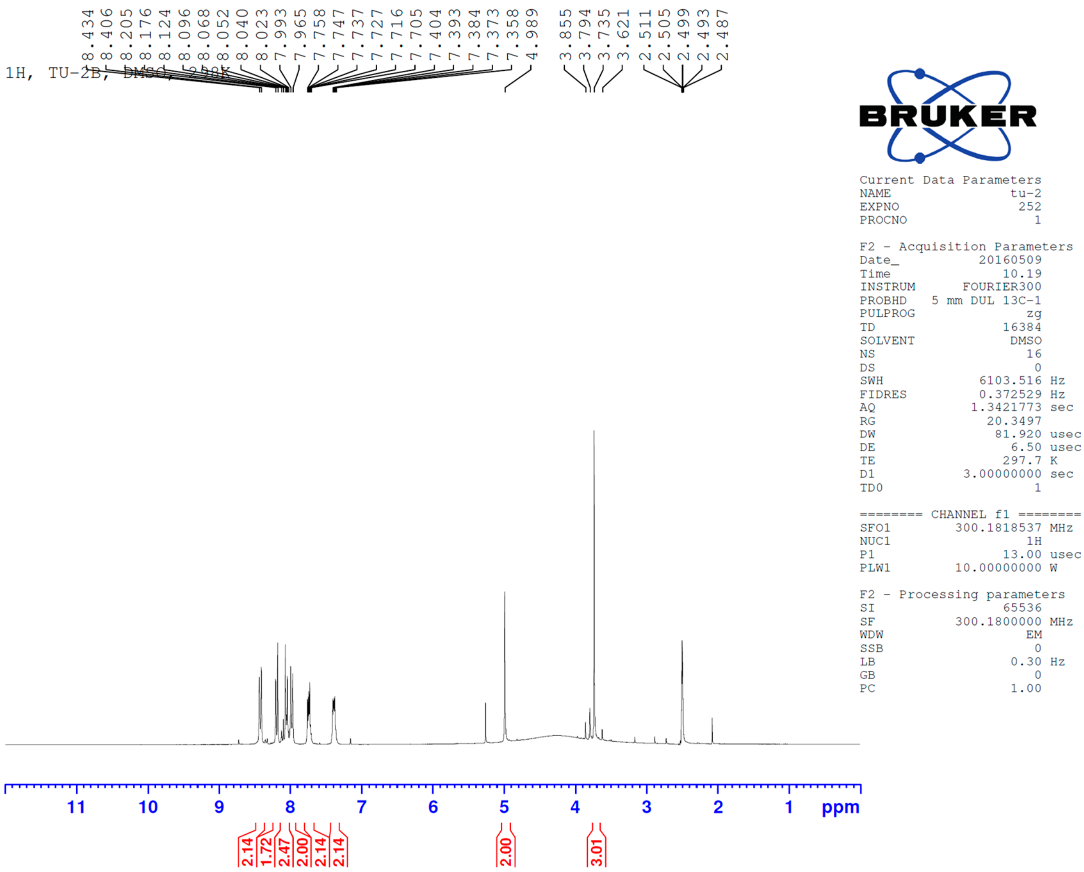

Figure S3. ^{1}H-NMR spectra of compound **5a**

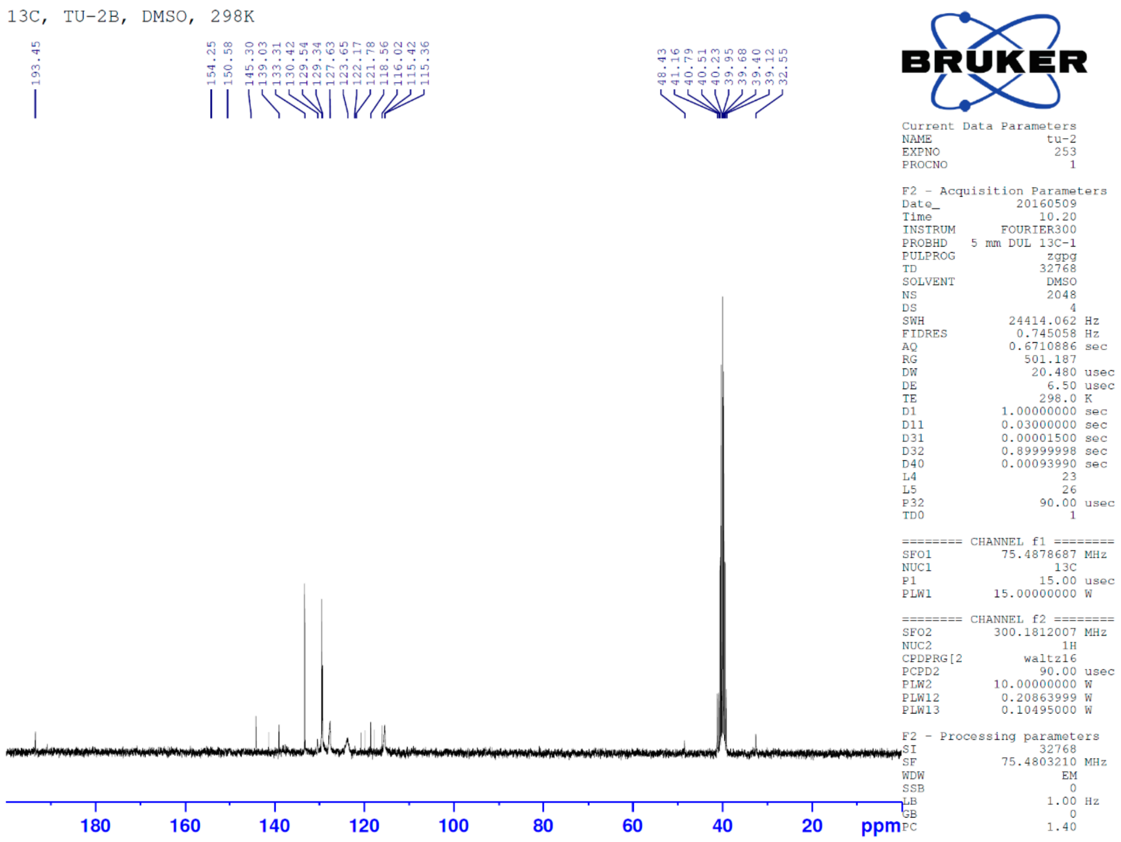

Figure S4. ^{13}C-NMR spectra of compound **5a**

Formula Predictor Report - TU-2B_6.lcd Page 1 of 1

Data File: C:\LabSolutions\Data\Analiz\uac\TU-2B_6.lcd

Elmt	Val.	Min	Max	Elmt	Val.	Min	Max	Elmt	Val.	Min	Max	Use Adduct
H	1	16	40	O	2	1	3	Cl	1	0	2	H
C	4	22	40	F	1	0	2	Br	1	0	1	
N	3	5	6	S	2	1	1					

Error Margin (ppm):	5	DBE Range:	17.0 - 30.0	Electron Ions:	both
HC Ratio:	unlimited	Apply N Rule:	yes	Use MSn Info:	no
Max Isotopes:	3	Isotope RI (%):	1.00	Isotope Res:	10000
MSn Iso RI (%):	10.00	MSn Logic Mode:	AND	Max Results:	500

Event#: 1 MS(E+) Ret. Time : 6.080 -> 6.080 Scan# : 913 -> 913

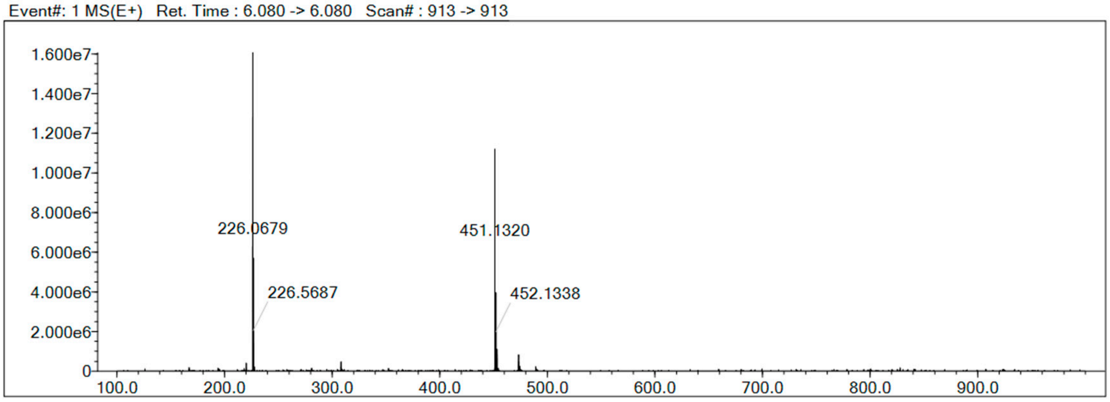

Measured region for 451.1320 m/z

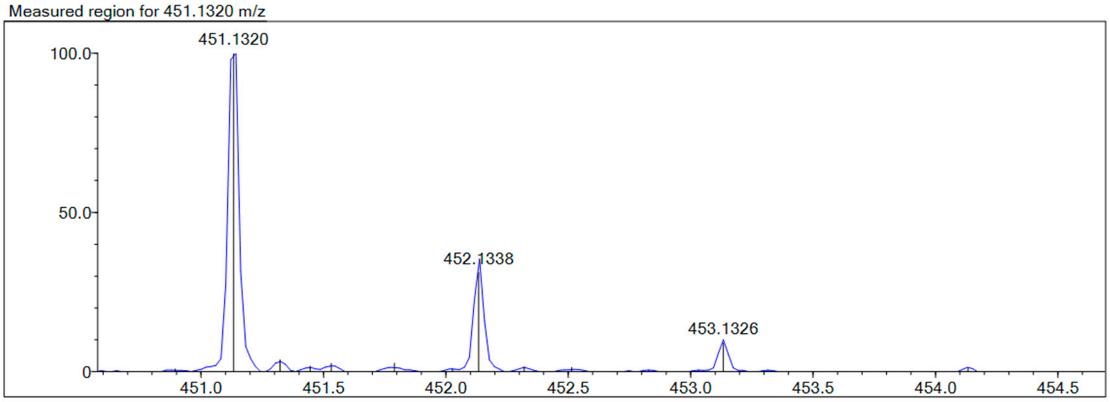

C25 H18 N6 O S [M+H]+ : Predicted region for 451.1336 m/z

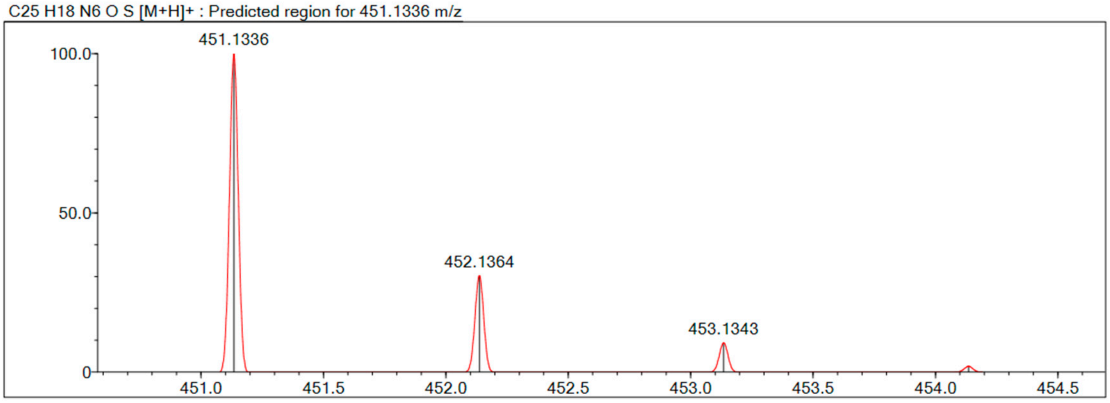

Rank	Score	Formula (M)	Ion	Meas. m/z	Pred. m/z	Df. (mDa)	Df. (ppm)	Iso	DBE
1	89.79	C25 H18 N6 O S	[M+H]+	451.1320	451.1336	-1.6	-3.55	95.91	20.0

Figure S5. Mass spectrum of compound **5a**

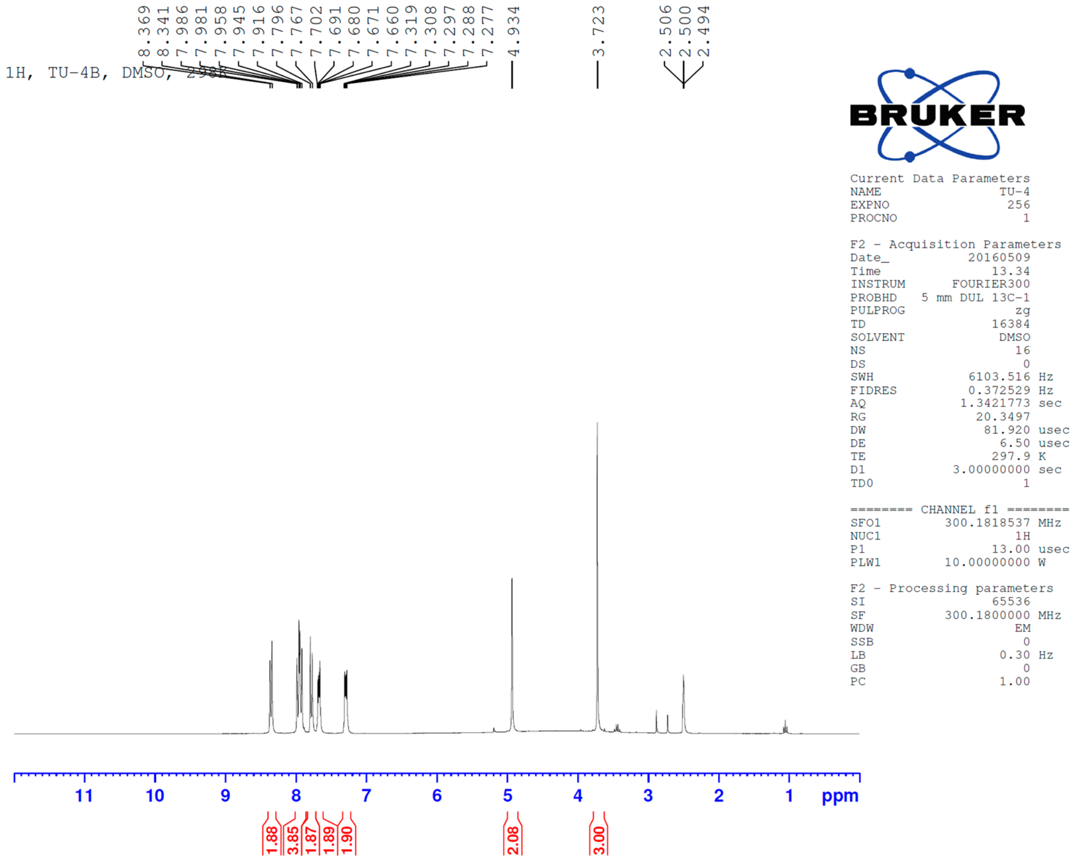

Figure S6. ^{1}H-NMR spectra of compound **5b**

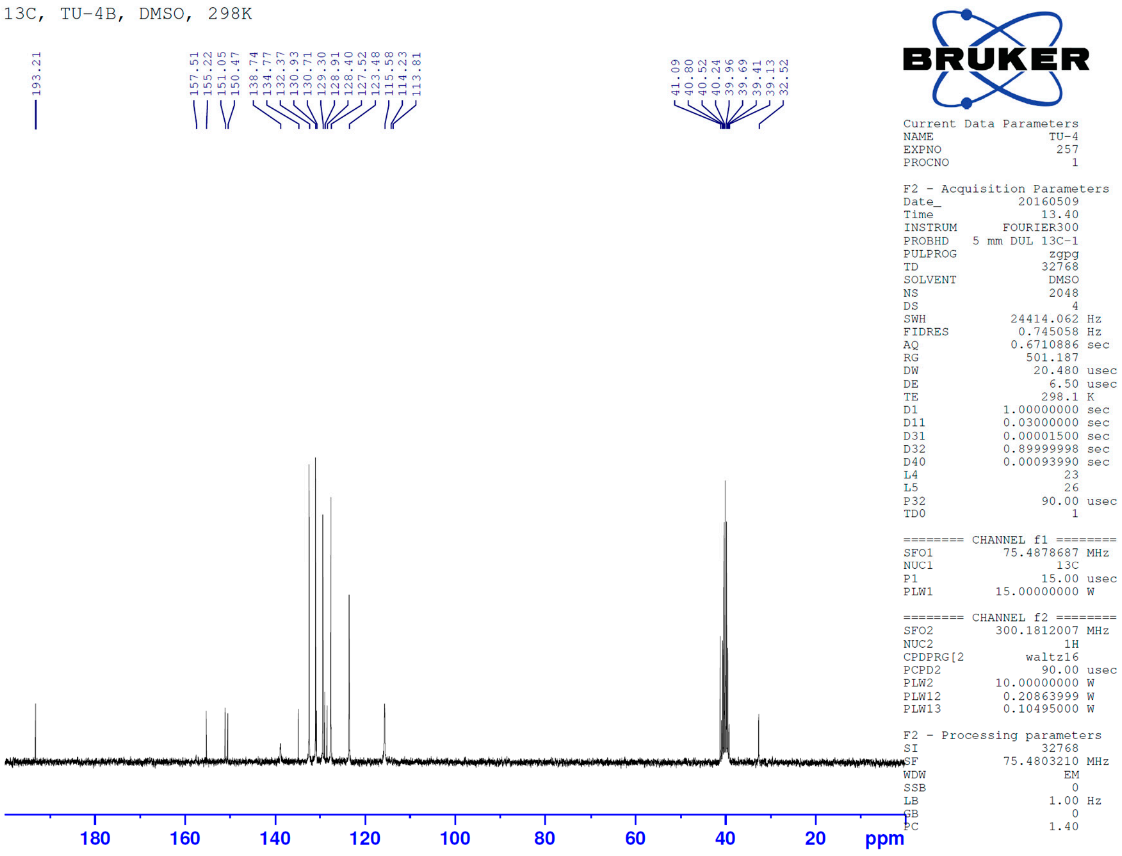

Figure S7. ^{13}C-NMR spectra of compound **5b**

Data File: C:\LabSolutions\Data\Analiz\uac\TU-4A_6.lcd

Elmt	Val.	Min	Max	Elmt	Val.	Min	Max	Elmt	Val.	Min	Max	Use Adduct
H	1	16	40	O	2	1	3	Cl	1	0	2	H
C	4	22	40	F	1	0	2	Br	1	0	1	
N	3	5	6	S	2	1	1					

Error Margin (ppm):	5	DBE Range:	17.0 - 30.0	Electron Ions:	both
HC Ratio:	unlimited	Apply N Rule:	yes	Use MSn Info:	no
Max Isotopes:	3	Isotope RI (%):	1.00	Isotope Res:	10000
MSn Iso RI (%):	10.00	MSn Logic Mode:	AND	Max Results:	500

Event#: 1 MS(E+) Ret. Time : 6.387 -> 6.387 Scan# : 959 -> 959

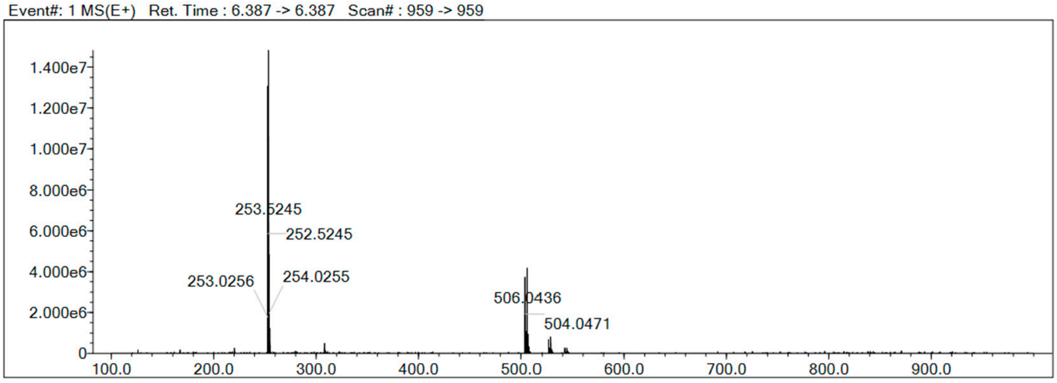

Measured region for 504.0471 m/z

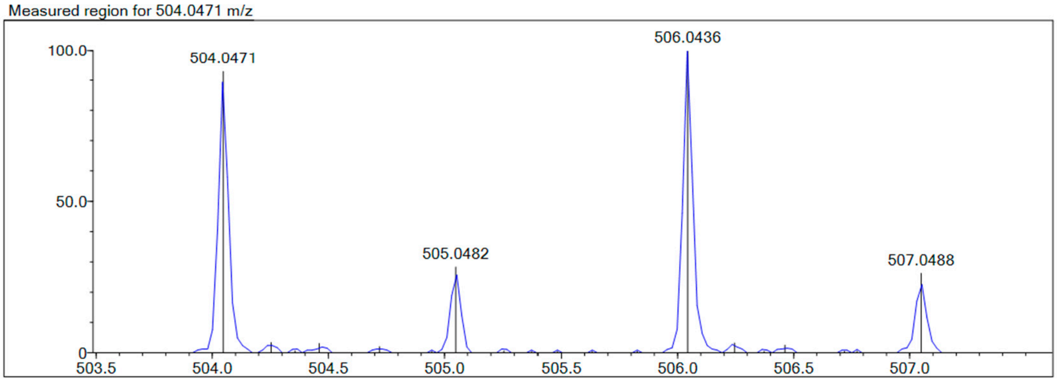

C24 H18 N5 O S Br [M+H]+ : Predicted region for 504.0488 m/z

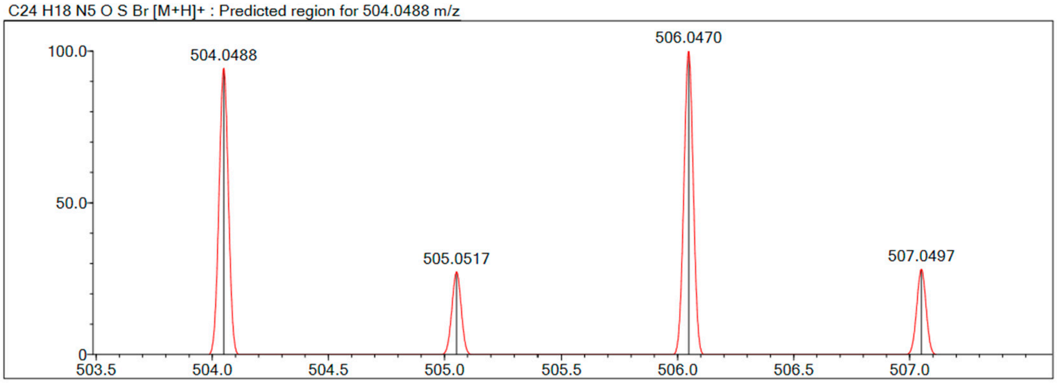

Rank	Score	Formula (M)	Ion	Meas. m/z	Pred. m/z	Df. (mDa)	Df. (ppm)	Iso	DBE
1	59.56	C24 H18 N5 O S Br	[M+H]+	504.0471	504.0488	-1.7	-3.37	63.31	18.0

Figure S8. Mass spectrum of compound **5b**

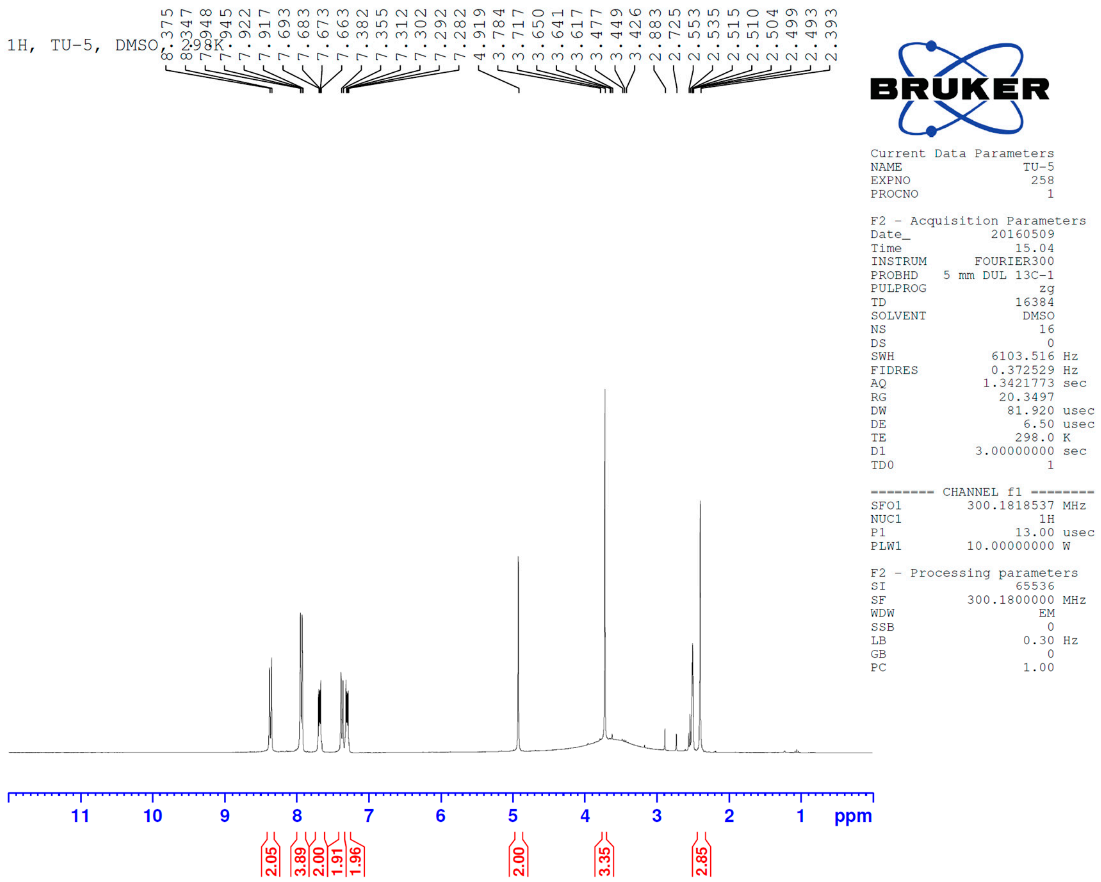

Figure S9. ^{1}H-NMR spectra of compound **5c**

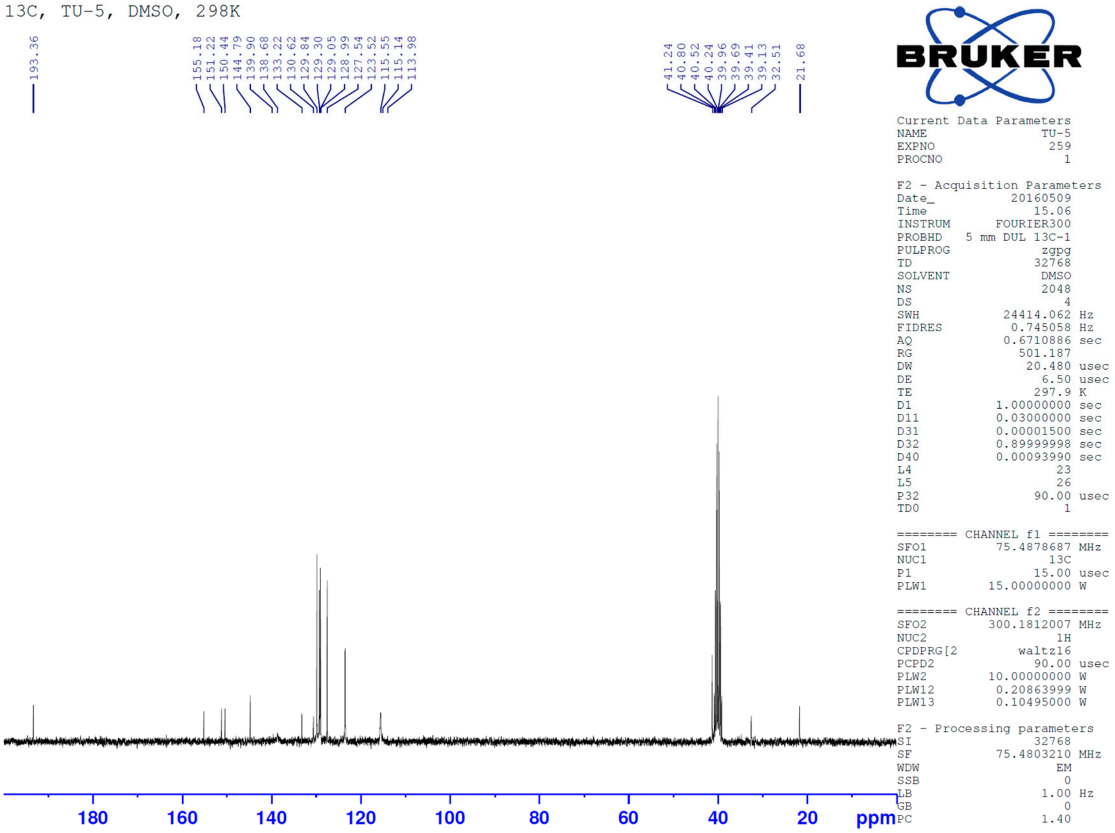

Figure S10. ^{13}C-NMR spectra of compound **5c**

Data File: C:\LabSolutions\Data\Analiz\uac\TU-5_8.lcd

Elmt	Val.	Min	Max	Elmt	Val.	Min	Max	Elmt	Val.	Min	Max	Use Adduct
H	1	16	40	O	2	1	3	Cl	1	0	2	H
C	4	22	40	F	1	0	2	Br	1	0	1	
N	3	5	6	S	2	1	1					

Error Margin (ppm):	5	DBE Range:	17.0 - 30.0	Electron Ions:	both
HC Ratio:	unlimited	Apply N Rule:	yes	Use MSn Info:	no
Max Isotopes:	3	Isotope RI (%):	1.00	Isotope Res:	10000
MSn Iso RI (%):	10.00	MSn Logic Mode:	AND	Max Results:	500

Event#: 1 MS(E+) Ret. Time : 6.240 -> 6.240 Scan# : 937 -> 937

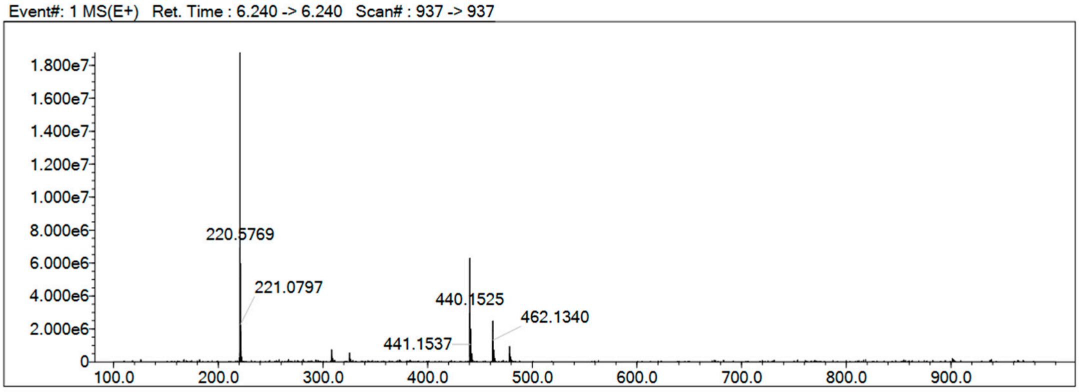

Measured region for 440.1525 m/z

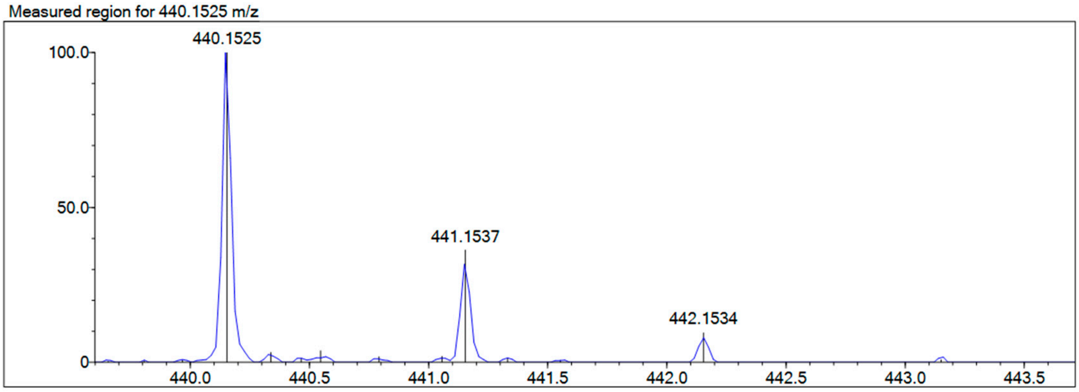

C25 H21 N5 O S [M+H]+ : Predicted region for 440.1540 m/z

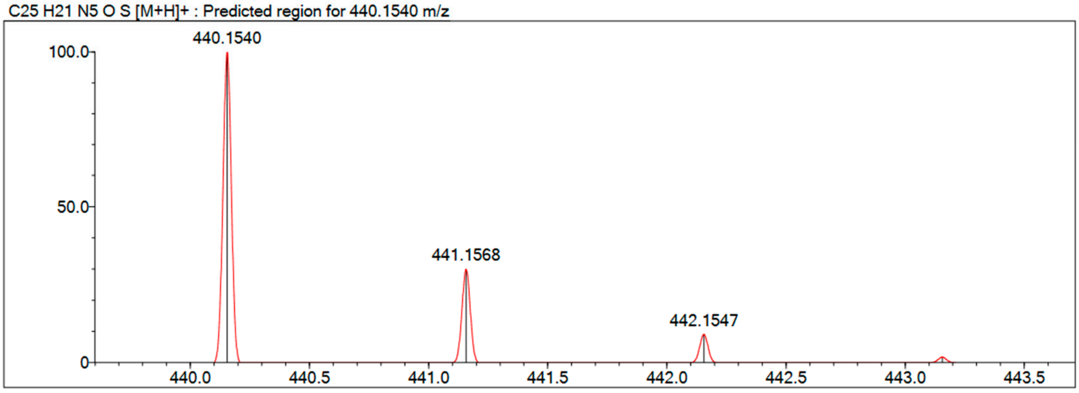

Rank	Score	Formula (M)	Ion	Meas. m/z	Pred. m/z	Df. (mDa)	Df. (ppm)	Iso	DBE
1	66.38	C25 H21 N5 O S	[M+H]+	440.1525	440.1540	-1.5	-3.41	70.64	18.0

Figure S11. Mass spectrum of compound **5c**

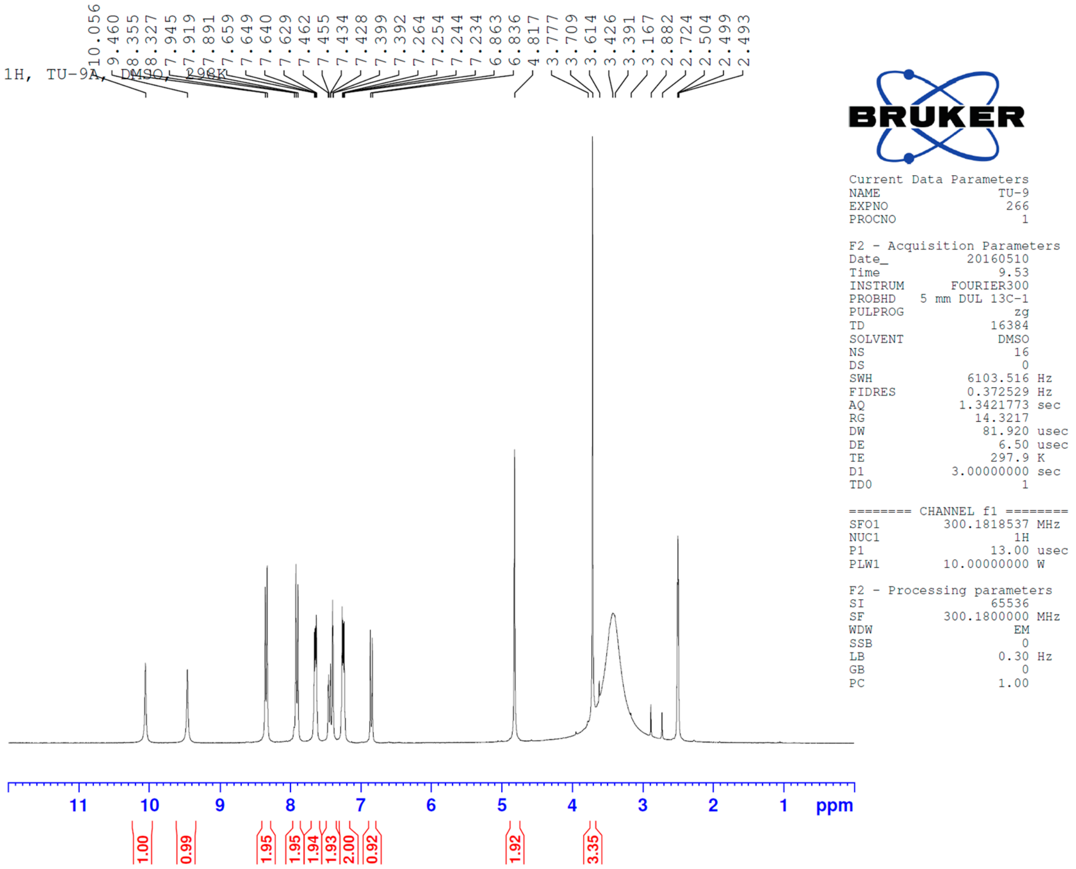

Figure S12. ^{1}H-NMR spectra of compound **5d**

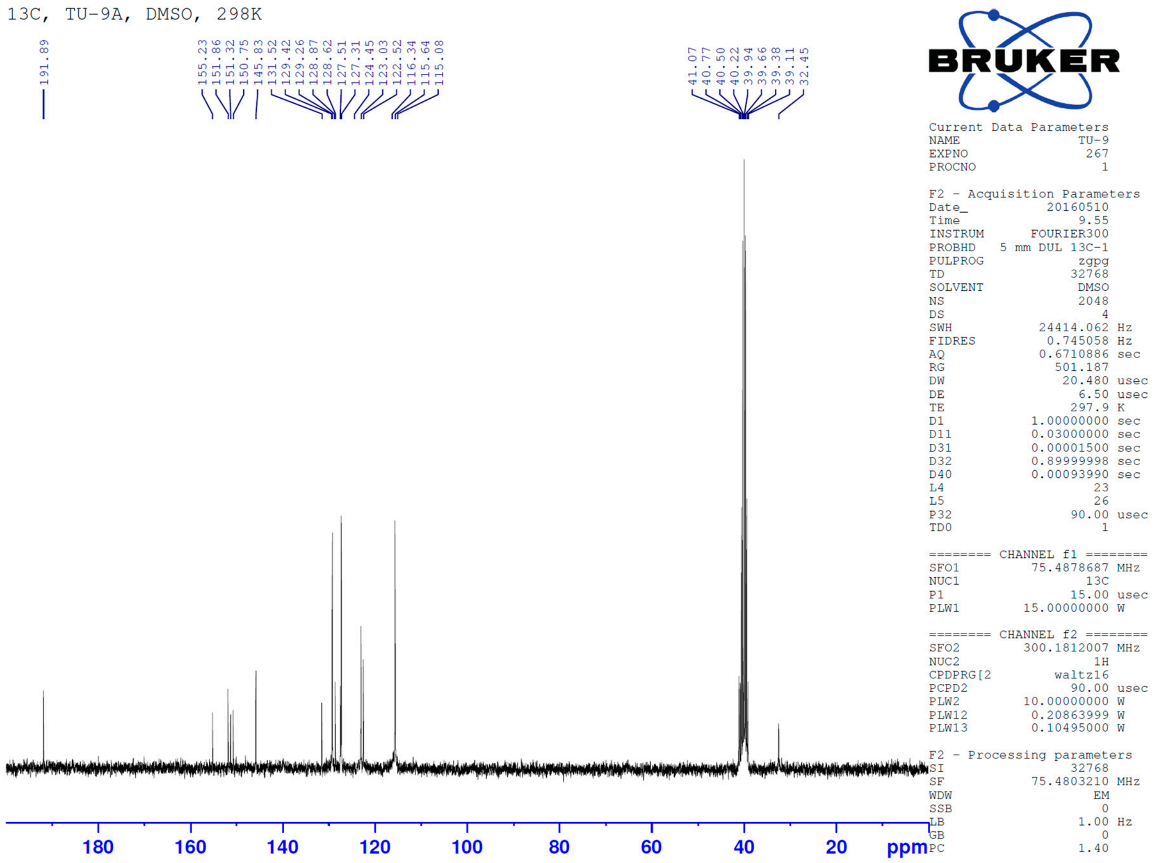

Figure S13. ^{13}C-NMR spectra of compound **5d**

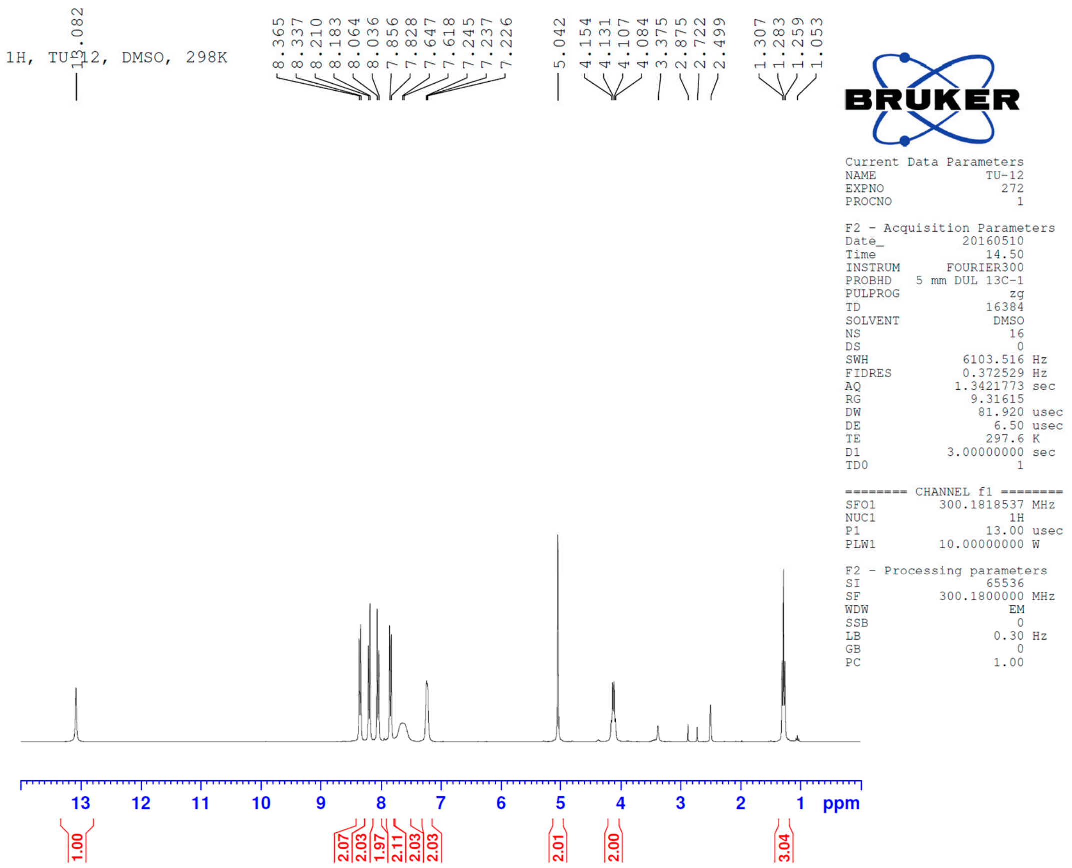

Figure S14. ^{1}H-NMR spectra of compound **5e**

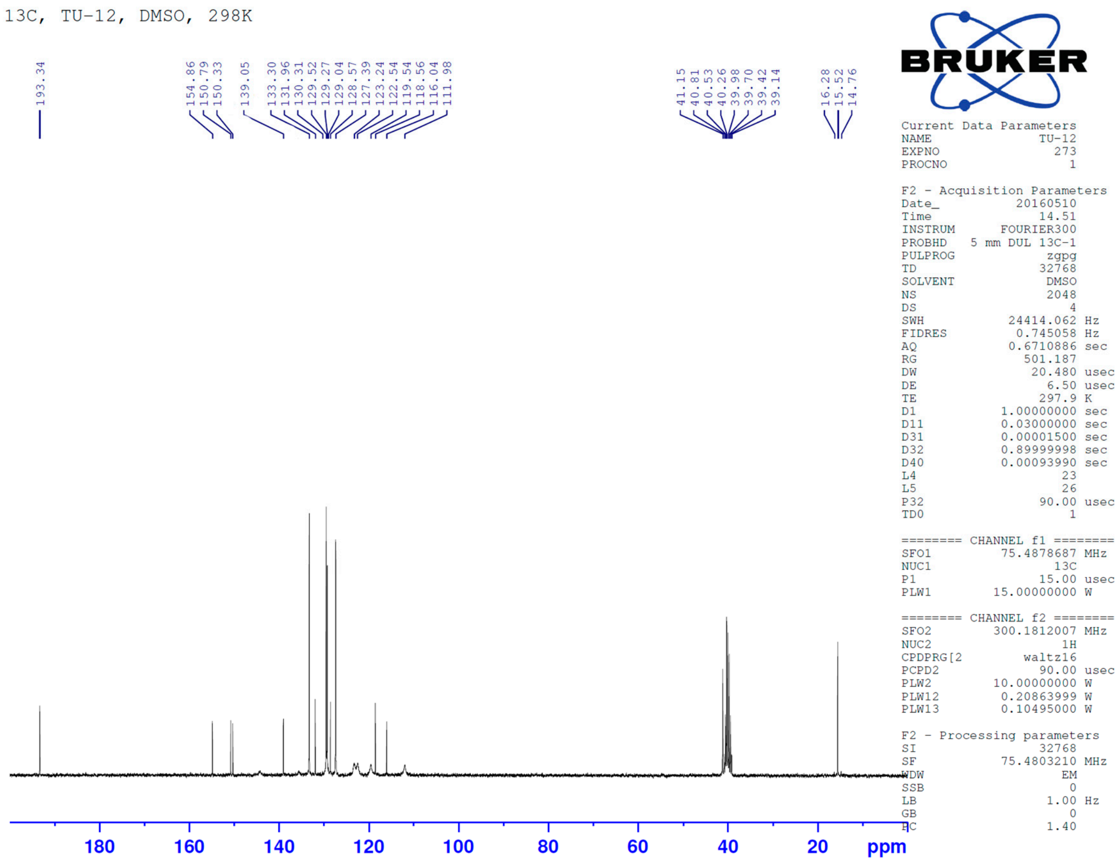

Figure S15. ^{13}C-NMR spectra of compound **5e**

Formula Predictor Report - TU-12_19.lcd

Data File: C:\LabSolutions\Data\Analiz\uac\TU-12_19.lcd

Elmt	Val.	Min	Max	Elmt	Val.	Min	Max	Elmt	Val.	Min	Max	Use Adduct
H	1	16	40	O	2	1	3	Cl	1	0	2	H
C	4	22	40	F	1	0	2	Br	1	0	1	
N	3	5	6	S	2	1	1					

Error Margin (ppm): 5
HC Ratio: unlimited
Max Isotopes: 3
MSn Iso RI (%): 10.00

DBE Range: 17.0 - 30.0
Apply N Rule: yes
Isotope RI (%): 1.00
MSn Logic Mode: AND

Electron Ions: both
Use MSn Info: no
Isotope Res: 10000
Max Results: 500

Event#: 1 MS(E+) Ret. Time : 6.160 -> 6.160 Scan# : 925 -> 925

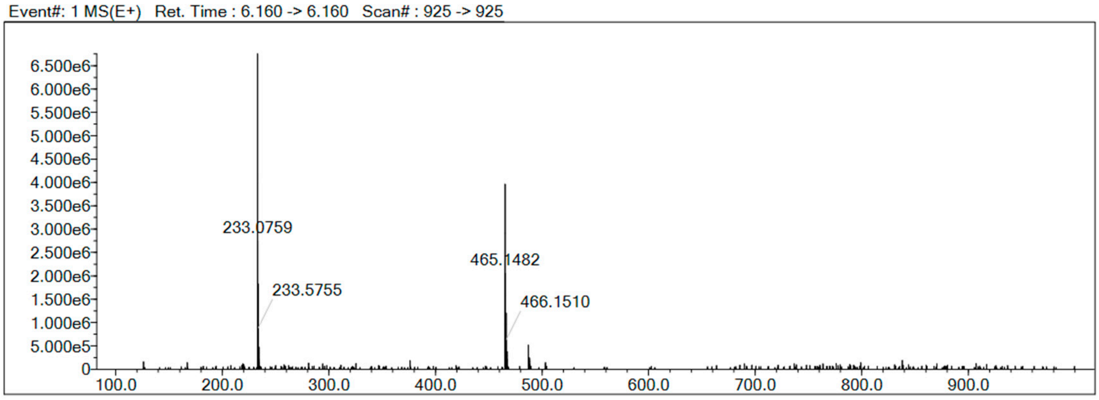

Measured region for 465.1482 m/z

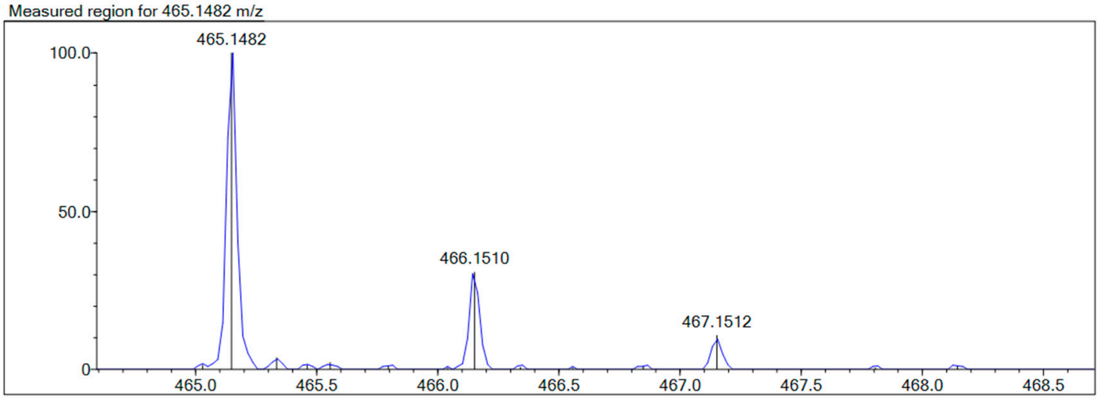

C26 H20 N6 O S [M+H]+ : Predicted region for 465.1492 m/z

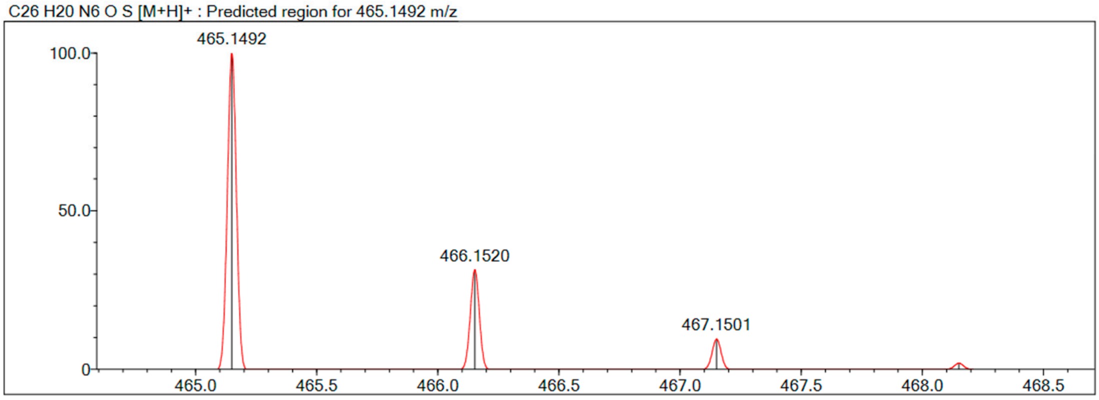

Rank	Score	Formula (M)	Ion	Meas. m/z	Pred. m/z	Df. (mDa)	Df. (ppm)	Iso	DBE
1	65.59	C26 H20 N6 O S	[M+H]+	465.1482	465.1492	-1.0	-2.15	67.53	20.0

Figure S16 Mass spectrum of compound **5e**

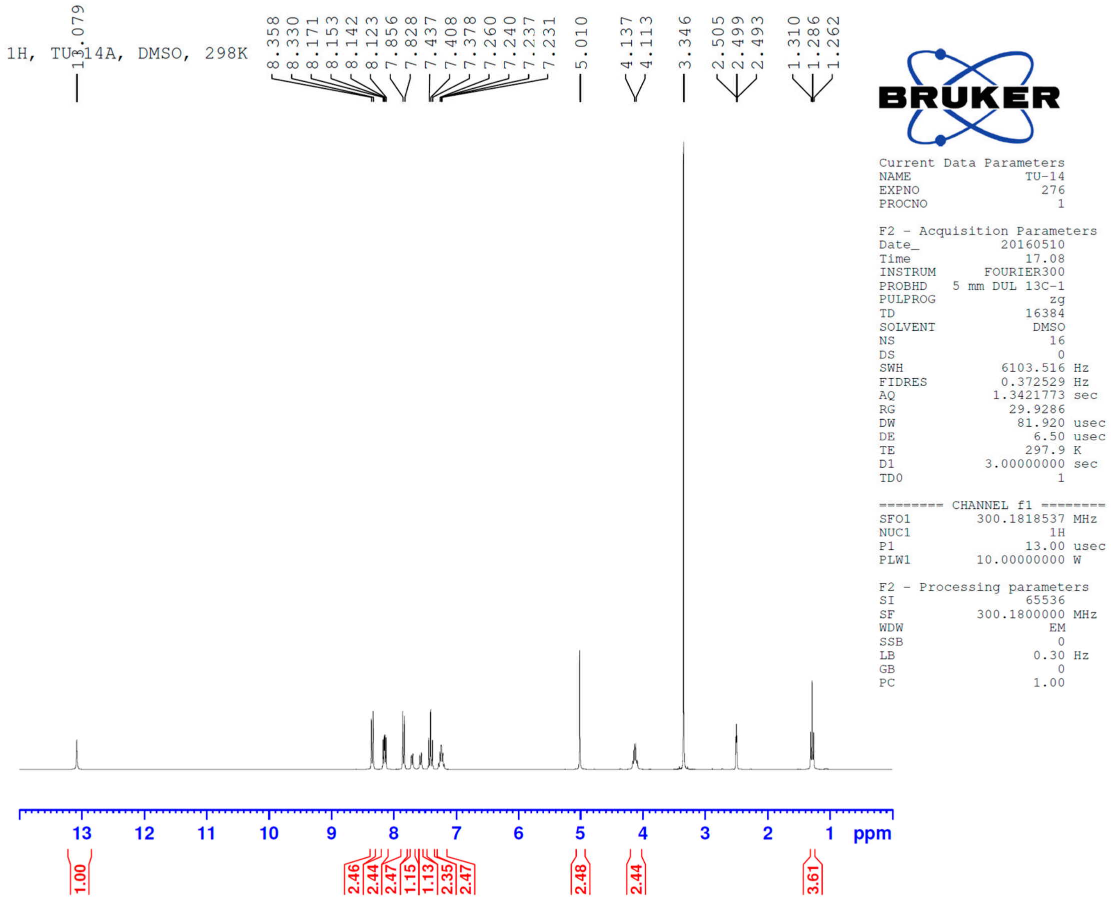

Figure S17. ^{1}H-NMR spectra of compound **5f**

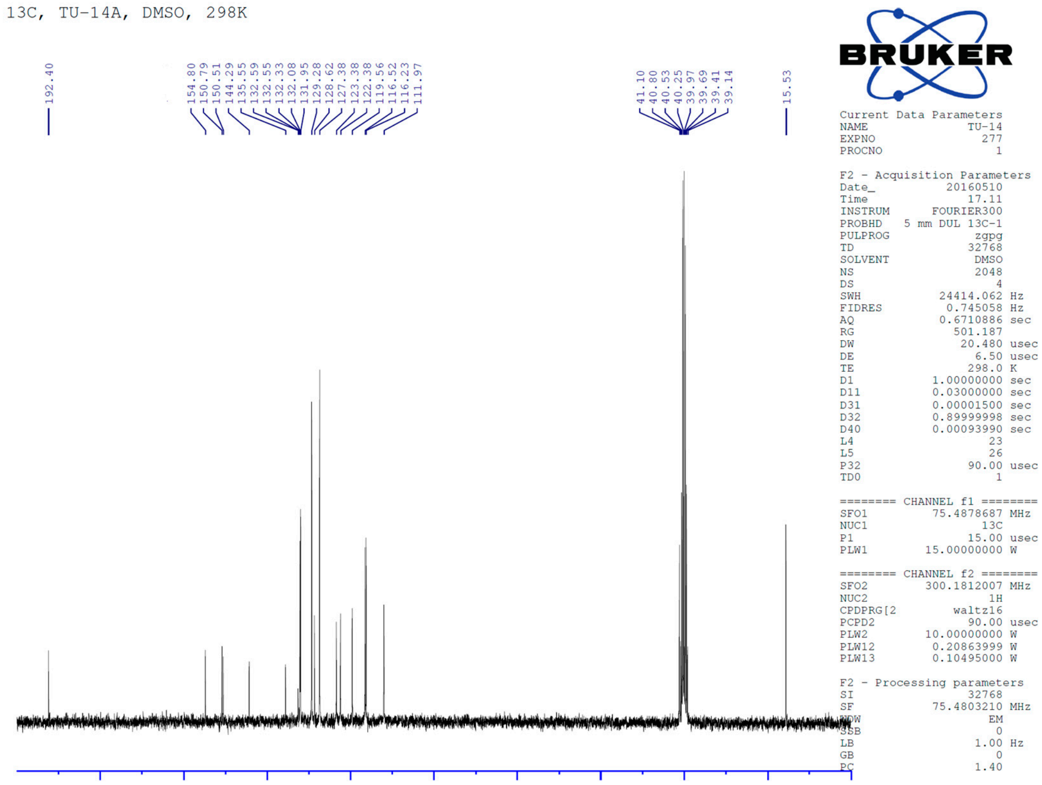

Figure S18. ^{13}C-NMR spectra of compound **5f**

Data File: C:\LabSolutions\Data\Analiz\uac\TU-14A_22.lcd

Elmt	Val.	Min	Max	Elmt	Val.	Min	Max	Elmt	Val.	Min	Max	Use Adduct
H	1	16	40	O	2	1	3	Cl	1	0	2	H
C	4	22	40	F	1	0	2	Br	1	0	1	
N	3	5	6	S	2	1	1					

Error Margin (ppm):	5	DBE Range:	17.0 - 30.0	Electron Ions:	both
HC Ratio:	unlimited	Apply N Rule:	yes	Use MSn Info:	no
Max Isotopes:	3	Isotope RI (%):	1.00	Isotope Res:	10000
MSn Iso RI (%):	10.00	MSn Logic Mode:	AND	Max Results:	500

Event#: 1 MS(E+) Ret. Time : 6.560 -> 6.560 Scan# : 985 -> 985

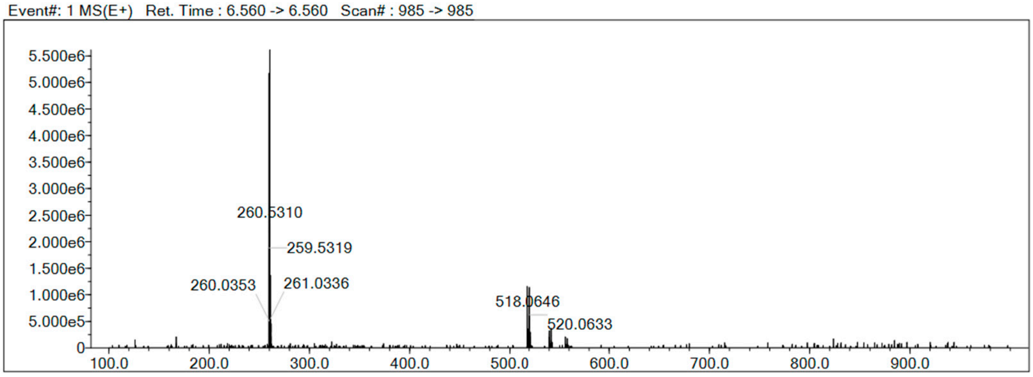

Measured region for 518.0646 m/z

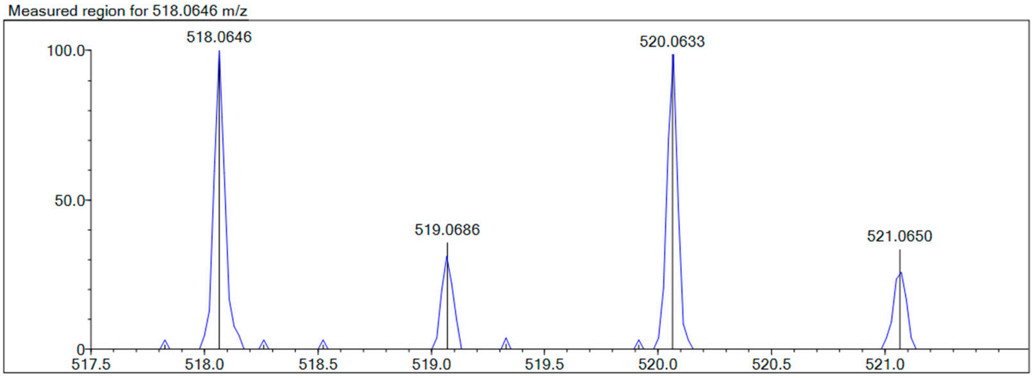

C25 H20 N5 O S Br [M+H]+ : Predicted region for 518.0645 m/z

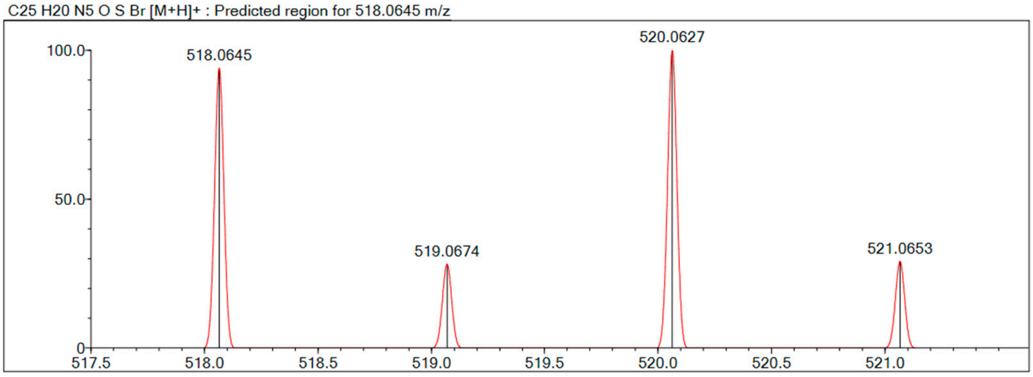

Rank	Score	Formula (M)	Ion	Meas. m/z	Pred. m/z	Df. (mDa)	Df. (ppm)	Iso	DBE
1	75.36	C25 H20 N5 O S Br	[M+H]+	518.0646	518.0645	0.1	0.19	75.36	18.0

Figure S19. Mass spectrum of compound **5f**

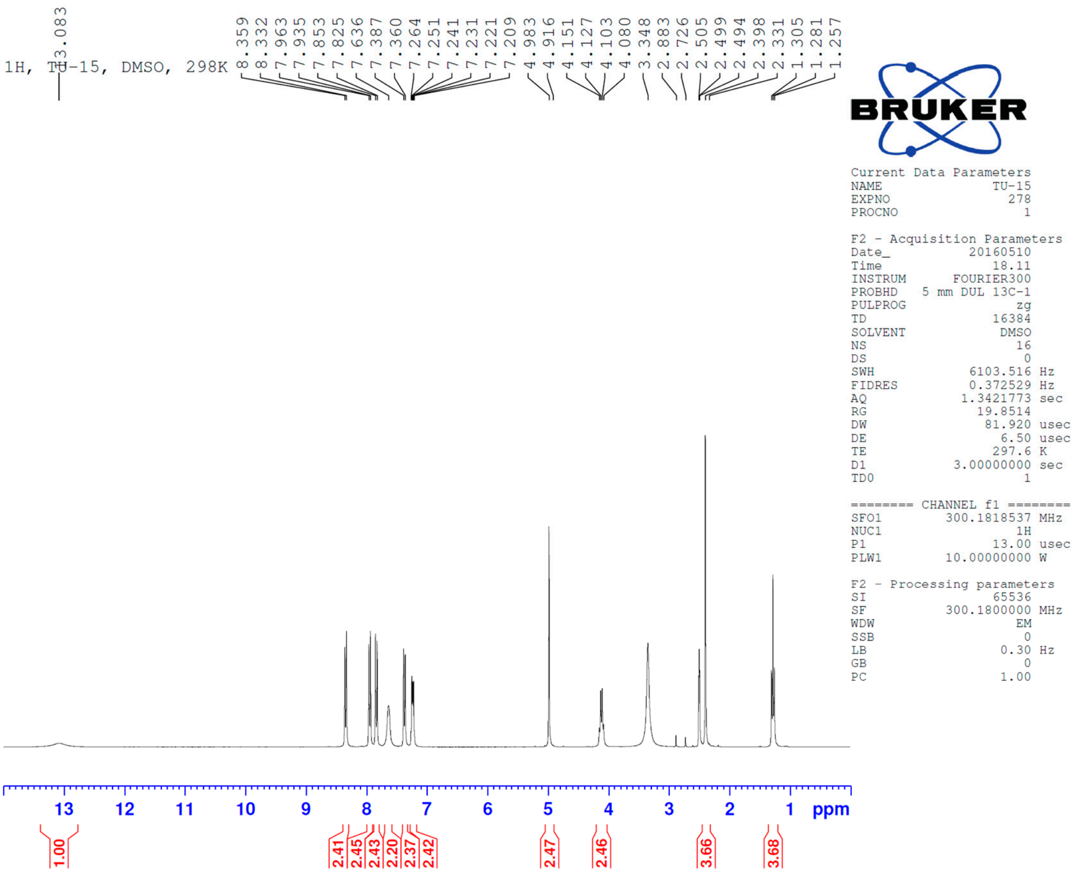

Figure S20. ^{1}H-NMR spectra of compound **5g**

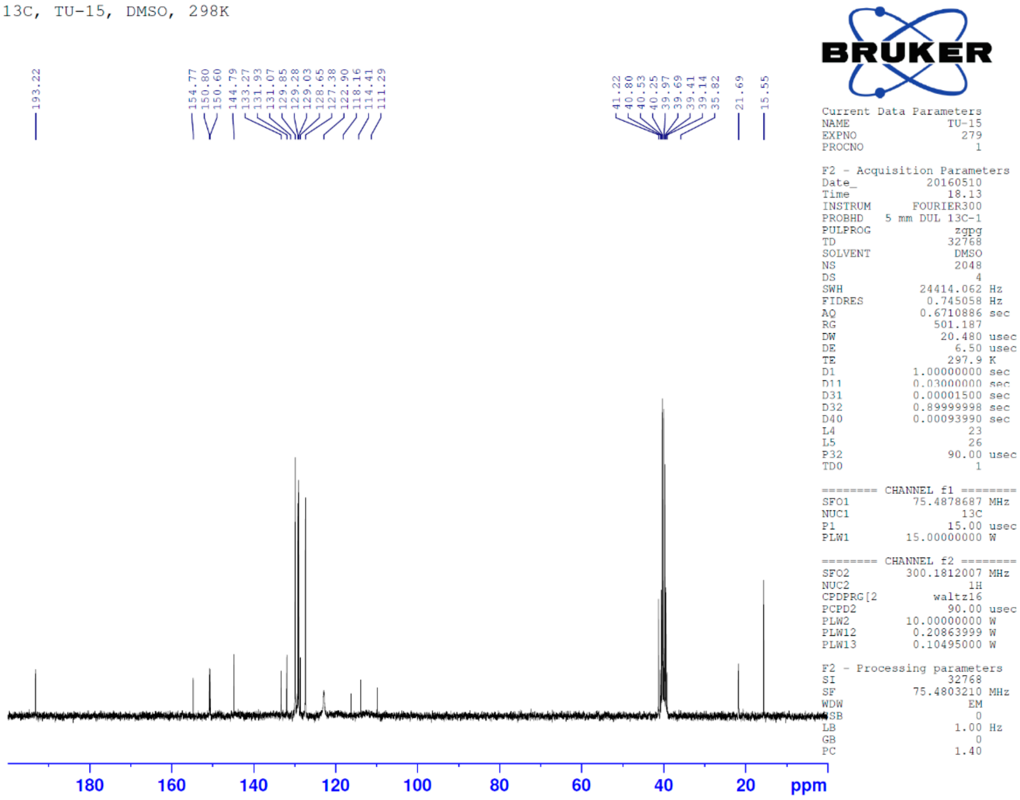

Figure S21. ^{13}C-NMR spectra of compound **5g**

Data File: C:\LabSolutions\Data\Analiz\uac\TU-15_24.lcd

Elmt	Val.	Min	Max	Elmt	Val.	Min	Max	Elmt	Val.	Min	Max	Use Adduct
H	1	16	40	O	2	1	3	Cl	1	0	2	H
C	4	22	40	F	1	0	2	Br	1	0	1	
N	3	5	6	S	2	1	1					

Error Margin (ppm): 5
HC Ratio: unlimited
Max Isotopes: 3
MSn Iso RI (%): 10.00

DBE Range: 17.0 - 30.0
Apply N Rule: yes
Isotope RI (%): 1.00
MSn Logic Mode: AND

Electron Ions: both
Use MSn Info: no
Isotope Res: 10000
Max Results: 500

Event#: 1 MS(E+) Ret. Time : 6.453 -> 6.453 Scan# : 969 -> 969

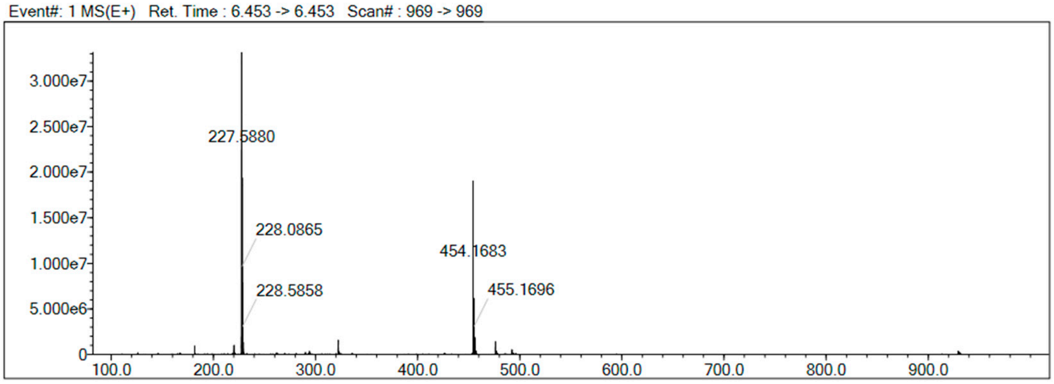

Measured region for 454.1683 m/z

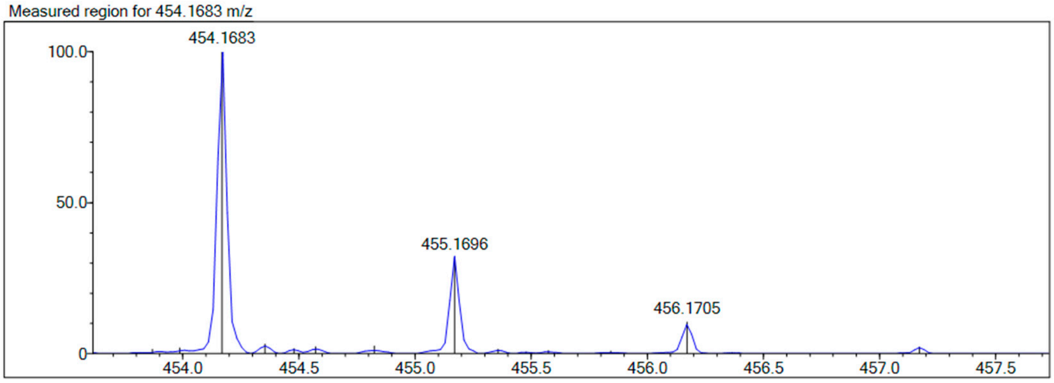

C26 H23 N5 O S [M+H]+ : Predicted region for 454.1696 m/z

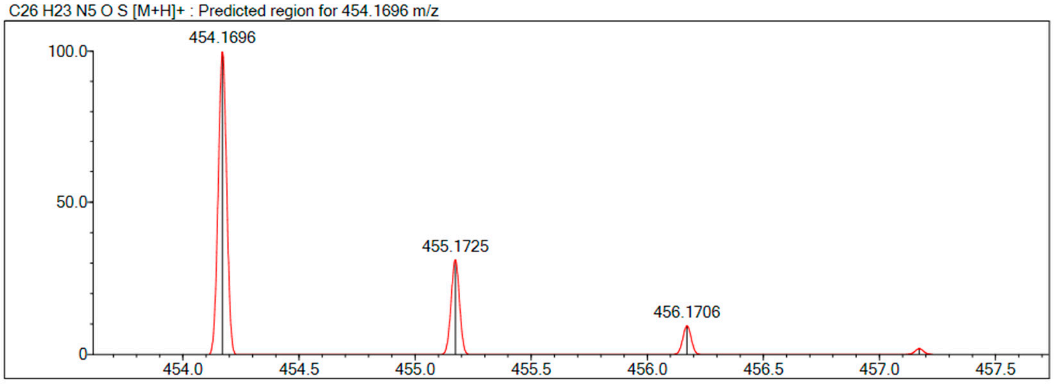

Rank	Score	Formula (M)	Ion	Meas. m/z	Pred. m/z	Df. (mDa)	Df. (ppm)	Iso	DBE
1	95.35	C26 H23 N5 O S	[M+H]+	454.1683	454.1696	-1.3	-2.86	100.00	18.0

Figure S22. Mass spectrum of compound **5g**

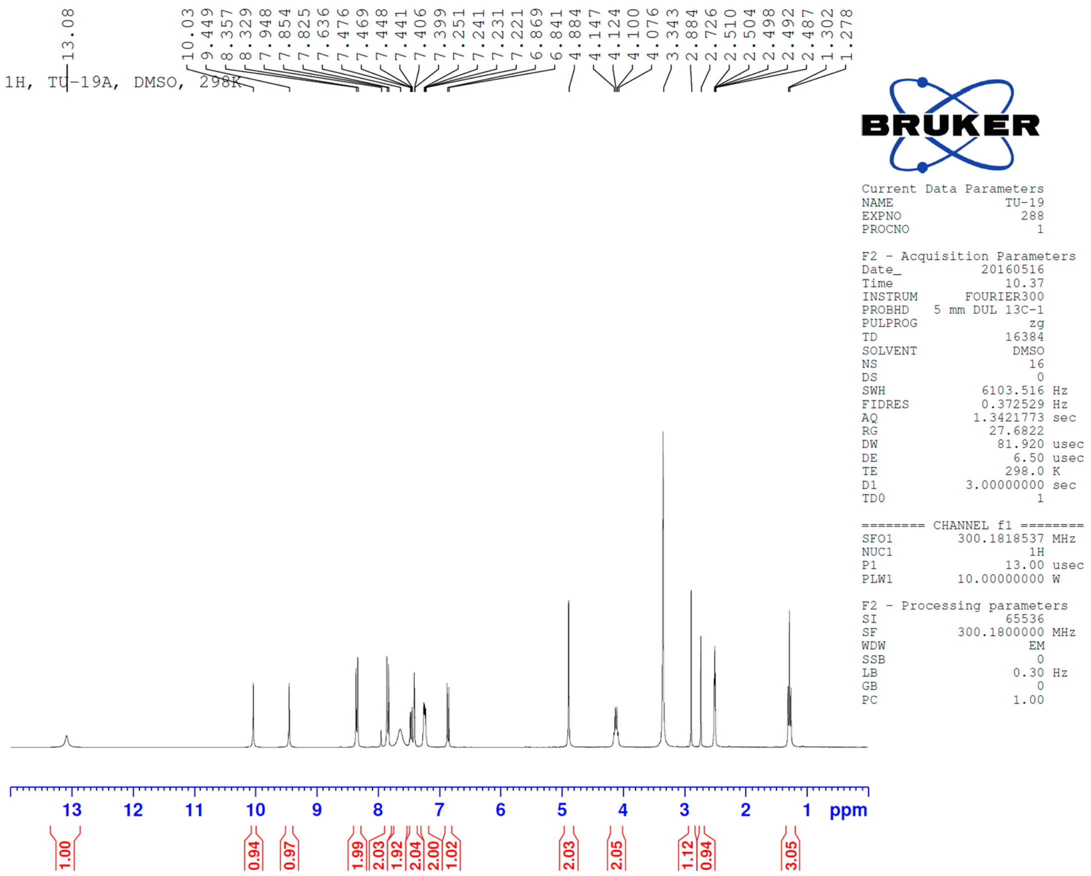

Figure S23. ^{1}H-NMR spectra of compound **5h**

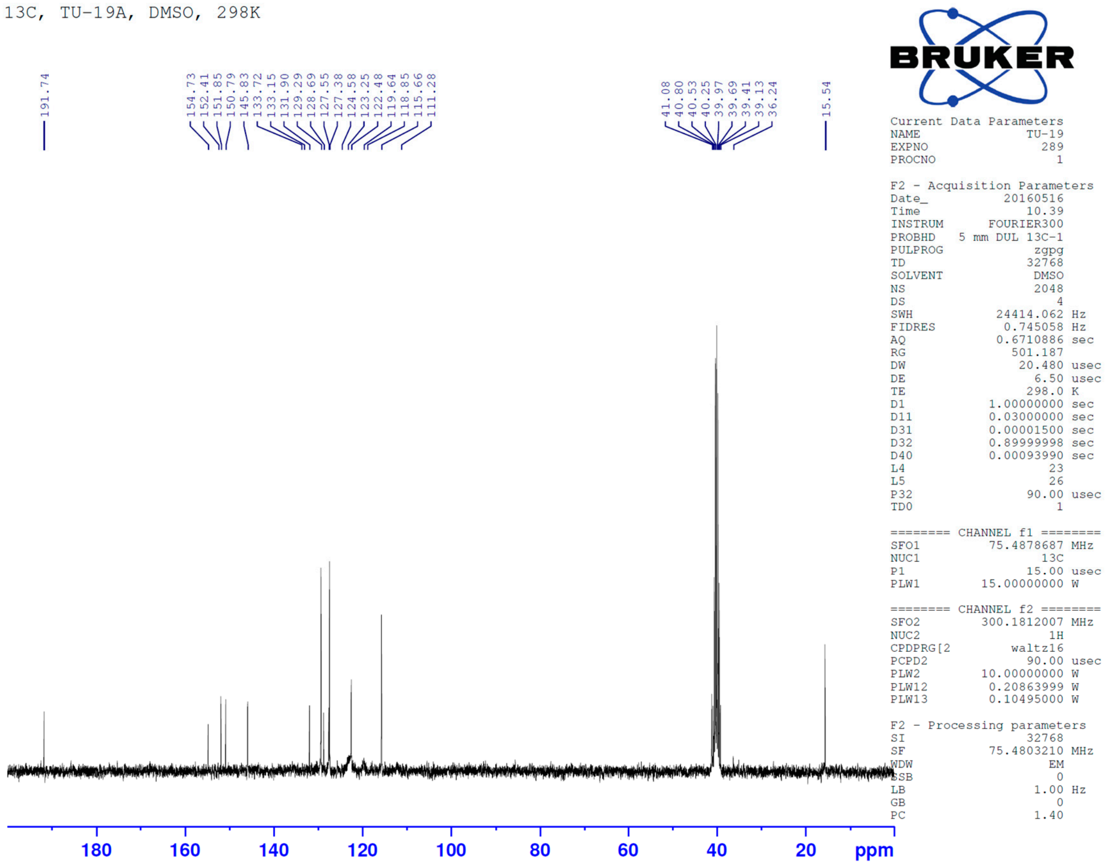

Figure S24. ^{13}C-NMR spectra of compound **5h**

Data File: C:\LabSolutions\Data\Analiz\uac\TU-19A_29.lcd

Elmt	Val.	Min	Max	Elmt	Val.	Min	Max	Elmt	Val.	Min	Max	Use Adduct
H	1	16	40	O	2	1	3	Cl	1	0	2	H
C	4	22	40	F	1	0	2	Br	1	0	1	
N	3	5	6	S	2	1	1					

Error Margin (ppm):	5	DBE Range:	17.0 - 30.0	Electron Ions:	both
HC Ratio:	unlimited	Apply N Rule:	yes	Use MSn Info:	no
Max Isotopes:	3	Isotope RI (%):	1.00	Isotope Res:	10000
MSn Iso RI (%):	10.00	MSn Logic Mode:	AND	Max Results:	500

Event#: 1 MS(E+) Ret. Time : 5.720 -> 5.720 Scan# : 859 -> 859

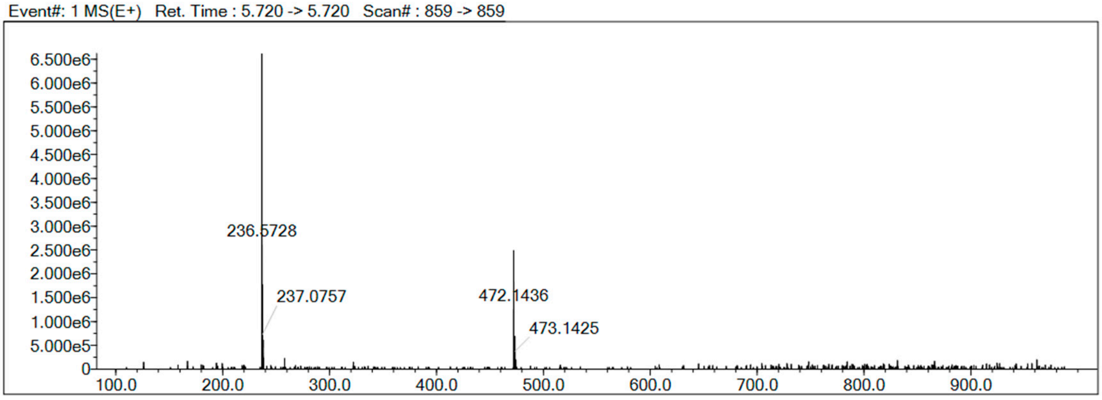

Measured region for 472.1436 m/z

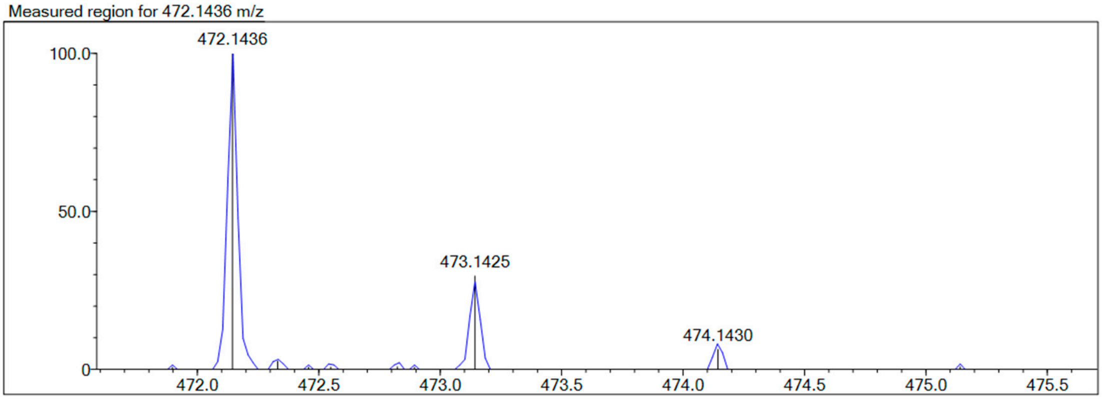

C25 H21 N5 O3 S [M+H]+ : Predicted region for 472.1438 m/z

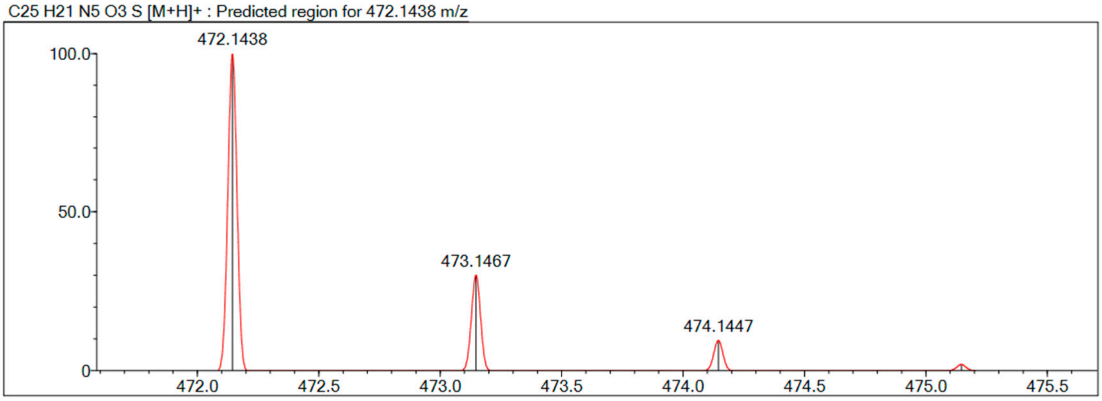

Rank	Score	Formula (M)	Ion	Meas. m/z	Pred. m/z	Df. (mDa)	Df. (ppm)	Iso	DBE
1	85.39	C25 H21 N5 O3 S	[M+H]+	472.1436	472.1438	-0.2	-0.42	85.39	18.0

Figure S25. Mass spectrum of compound **5h**
